# The atypical Rab GTPase associated with Parkinson’s disease, Rab29, is localized to membranes

**DOI:** 10.1016/j.jbc.2022.102499

**Published:** 2022-09-16

**Authors:** Yuki Nagai-Ito, Lejia Xu, Kyohei Ito, Yotaro Kajihara, Genta Ito, Taisuke Tomita

**Affiliations:** 1 Laboratory of Neuropathology and Neuroscience, Graduate School of Pharmaceutical Sciences, The University of Tokyo, Tokyo, Japan; 2 Department of Biomolecular Chemistry, Faculty of Pharma-Science, Teikyo University, Tokyo, Japan; 3 Social Cooperation Program of Brain and Neurological Disorders, Graduate School of Pharmaceutical Sciences, The University of Tokyo, Tokyo, Japan

**Keywords:** Rab, Parkinson’s disease, membrane trafficking, subcellular fractionation, protein isoprenylation, BME, β-mercaptoethanol, cDNA, complementary DNA, DKO, double knockout, dox, doxycycline, ER, endoplasmic reticulum, GAP, GTPase-activating protein, GDI, GDP-dissociation inhibitor, GEF, guanine nucleotide exchange factor, GGPP, geranylgeranyl diphosphate, gRNA, guide RNA, PD, Parkinson’s disease, RE buffer, Rab extraction buffer, REP, Rab escort protein, TGN, *trans*-Golgi network, TX114, Triton X-114, v/v, volume/volume

## Abstract

Several genetic studies have shown that the small GTPase Rab29 is involved in the pathogenesis of Parkinson’s Disease (PD). It has also been shown that overexpression of Rab29 increases the activity of leucine-rich repeat kinase 2, a protein kinase often mutated in familial PD, although the mechanism underlying this activation remains unclear. Here, we employed biochemical analyses to characterize the localization of Rab29 and found that, unlike general Rab proteins, Rab29 is predominantly fractionated into the membrane fraction by ultracentrifugation. We also found that Rab29 is resistant to extraction from membranes by GDP-dissociation inhibitors (GDIs) *in vitro*. Furthermore, Rab29 failed to interact with GDIs, and its membrane localization was not affected by the knockout of GDIs in cells. We show that the knockout of Rab geranylgeranyltransferase decreased the hydrophobicity of Rab29, suggesting that Rab29 is geranylgeranylated at its carboxyl terminus as is with typical Rab proteins. Notably, we demonstrated that membrane-bound Rab29 retains some hydrophilicity, indicating that mechanisms other than geranylgeranylation might also be involved in the membrane localization of Rab29. Taken together, these findings uncover the atypical nature of Rab29 among Rab proteins, which will provide important clues for understanding how Rab29 is involved in the molecular pathomechanism of PD.

Parkinson’s disease (PD) is one of the major neurodegenerative diseases of adult-onset. The cardinal symptoms of PD include resting tremor, muscle rigidity, akinesia, and postural instability caused by selective degeneration of dopaminergic neurons in the substantia nigra in the midbrain. The majority of PD is sporadic, and genome-wide association studies have identified loci associated with the development of sporadic PD. One of the loci associated with an increased risk of developing PD is *PARK16*, which contains three genes, namely *NUCKS1*, *RAB29*, and *SLC41A1* (1, 2, 3, 4). Among these genes, *RAB29* has attracted much attention as Rab29 was found to be in the same pathway as leucine-rich repeat kinase 2, a Ser/Thr protein kinase genetically linked with both familial (PARK8) as well as sporadic PD (5, 6, 7, 8, 9, 10, 11). Furthermore, several studies have shown that single nucleotide polymorphisms in the putative promoter region of *RAB29* (rs1572931 and rs823144) are associated with a decreased risk of developing sporadic PD (12, 13, 14, 15), underscoring the importance of Rab29 in the pathogenesis of PD.

Rab29 belongs to the Rab GTPase family consisting of more than 60 member proteins in humans. It has been well established that Rab proteins are involved in the regulation of intracellular vesicle trafficking. After ribosomal translation, partially folded Rab polypeptides are recognized by Rab escort proteins (REPs) for proper folding and then recruited to Rab geranylgeranyltransferase (RabGGTase) to be geranylgeranylated at the carboxyl-terminal cysteine(s) (16, 17, 18, 19, 20). Once geranylgeranylated, Rab proteins are inserted into lipid bilayers due to the hydrophobicity conferred by geranylgeranylation. Rab proteins then begin to cycle between donor and acceptor membranes by changing their bound guanine nucleotide with the help of guanine nucleotide exchange factors (GEFs) and GTPase-activating proteins (GAPs). Upon reaching acceptor membranes, Rab proteins interact with GAPs and are inactivated by hydrolyzing bound GTP to GDP. Inactivated Rab proteins are extracted from membranes to the cytosol by GDP-dissociation inhibitors (GDIs) and recycled back to donor membranes, where GEFs exist for activation (21). Although GEFs and GAPs for Rab29 are not known, it has been documented in the literature that Rab29 is atypical in that it does not form a complex with GDIs in contrast to typical Rab proteins including Rab8 and that it fails to undergo efficient geranylgeranylation in a cell-free experiment (22). However, a detailed analysis of the membrane localization and geranylgeranylation of Rab29 has not been performed.

In this report, we comprehensively examined the membrane localization, the hydrophobicity, and the GDI-dependent membrane extraction of Rab29 *in vitro* and in cultured cells in comparison with other Rab proteins. We herein show that Rab29 is hydrophobic to a similar extent as other Rab proteins but is not extracted from membranes by GDIs and exists predominantly as a membrane-bound form, unlike other Rab proteins. We also show that the membrane-bound Rab29 retains some hydrophilicity, suggesting that other mechanisms besides geranylgeranylation are involved in the membrane localization of Rab29.

## Results

### Endogenous Rab29 is predominantly fractionated into the membrane fraction

**Figure 1:**
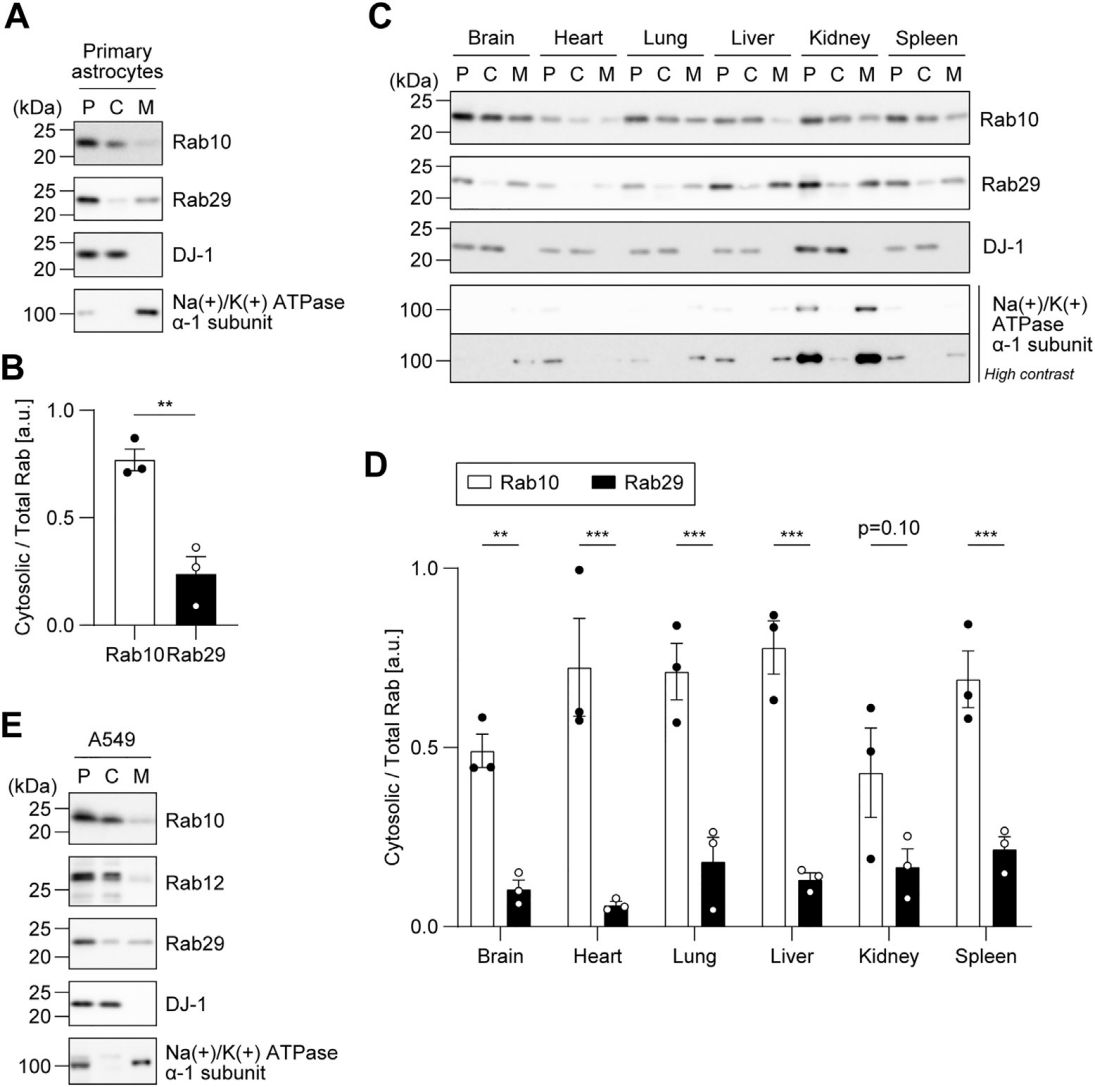
**Biochemical fractionation of endogenous Rab29.** Post-nuclear supernatants of (*A*) mouse primary glial cultures, (*C*) rat tissues (brain, heart, lung, liver, kidney, and spleen), and (*E*) A549 cells were fractionated by ultracentrifugation into the cytosol and membrane fractions. Representative immunoblots of three independent experiments with the indicated antibodies are shown. DJ-1 was used as a representative cytosolic protein, whereas Na+/K+ ATPase α-1 subunit was used as a membrane protein in (*A*, *C*, and *E*). *B* and *D*, the band intensity of Rab10 and Rab29 was quantified, and the ratio of cytosolic Rab to total Rab (cytosol + membrane) was calculated. The circles, the bars, and the error bars in the graphs represent individual values, the mean values, and their standard errors, respectively. ∗∗*p* < 0.01, ∗∗∗*p* < 0.001. The *p*-values and statistical tests used are summarized in Table S1.

To biochemically examine whether endogenous Rab29 exists in the cytosol or membrane fraction, we fractionated homogenates prepared from mouse primary glial cells into the cytosol and membrane fractions by ultracentrifugation. Rab10 was mainly fractionated into the cytosol fraction, whereas Rab29 was predominantly fractionated into the membrane fraction (Fig. 1*A*). The amount of endogenous Rab29 in the cytosol fraction was significantly lower than that of endogenous Rab10 (Fig. 1*B*). We then fractionated various rat tissue homogenates by ultracentrifugation. The majority of endogenous Rab29 was fractionated into the membrane fraction in all tissues, while Rab10 was mainly fractionated into the cytosol fraction (Fig. 1, *C* and *D*). In addition, fractionation of A549 cells showed that while Rab10 and Rab12 are mostly cytosolic, a larger part of Rab29 was fractionated into the membrane than into the cytosol, approximately at the ratio of 2 to 1 (Fig. 1*E*). These results suggested that although the degree varies among cell lines and organs, endogenous Rab29 is mainly fractionated into the membrane fraction, unlike Rab10.

As newly synthesized Rab proteins are first captured by REPs in the cytosol before undergoing lipid modification, Rab29 existing in the cytosol might represent newly synthesized molecules. To test this hypothesis, we treated cells with cycloheximide for 48 h to block protein synthesis and deplete the newly synthesized molecules. The cells were then fractionated by ultracentrifugation. However, the ratio of cytosolic to total Rab proteins was not significantly changed upon treatment with cycloheximide (Fig. S1), suggesting that Rab29 existing in the cytosol does not represent newly synthesized molecules and that the “age” of Rab10 and Rab29 has no significant effect on their membrane association.

### Rab29 is resistant to the extraction from membranes by GDIs

**Figure 2:**
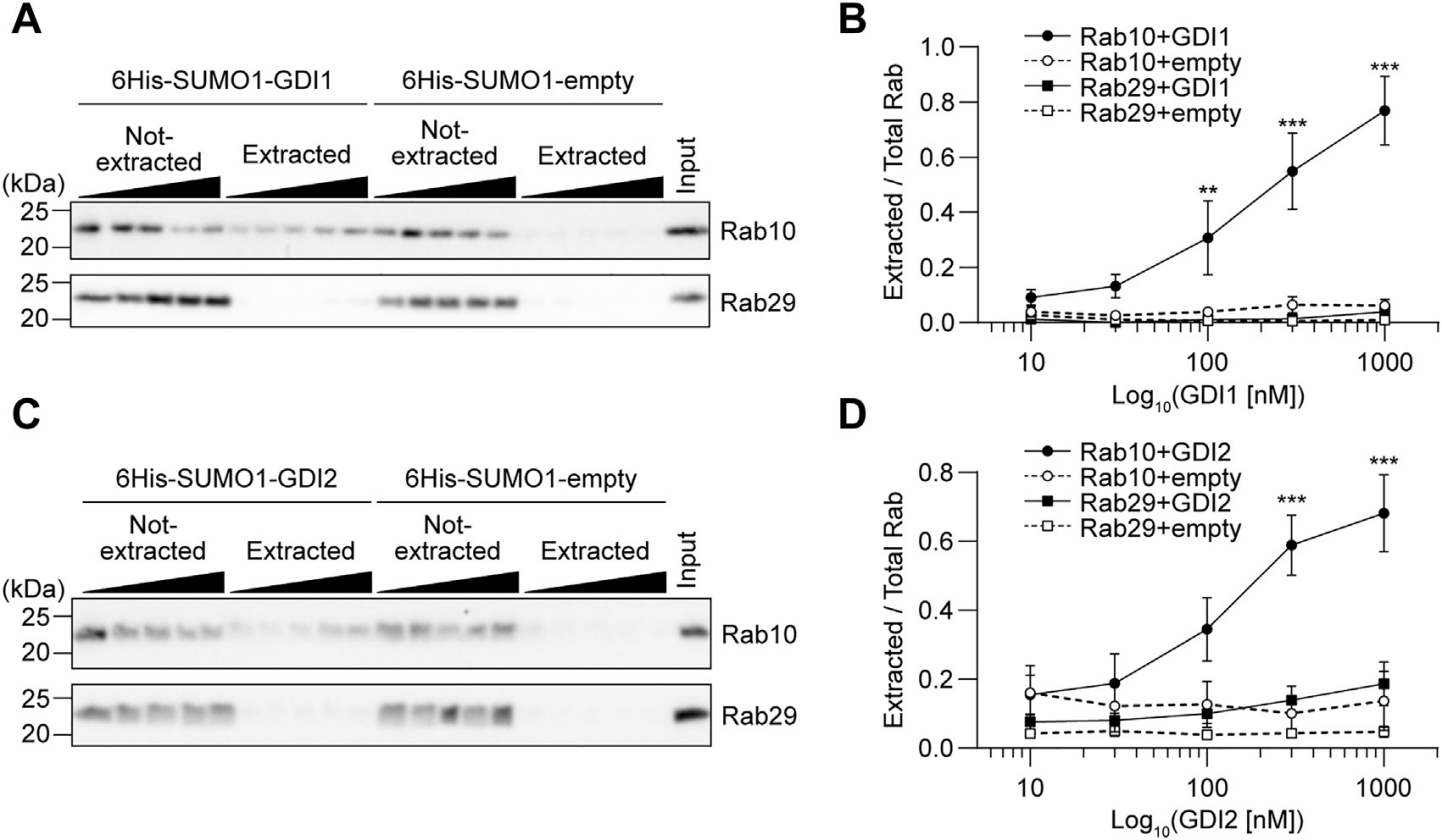
***In vitro* extraction assay of Rab10 and Rab29 by GDI1/2.** Membrane fractions of HEK293A cells were incubated in the presence of the indicated concentration of recombinant 6His-SUMO1-GDI1 or 6His-SUMO1-GDI2, and the assay mixtures were fractionated into the extracted and not-extracted fractions by ultracentrifugation. Each experiment was carried out side-by-side with the 6His-SUMO1-empty control protein to exclude the effect of the 6His-SUMO1 tag. *A* and *C*, representative immunoblots of three independent experiments with the indicated antibodies are shown. *B* and *D*, the band intensities of Rab10 and Rab29 were quantified, and the ratio of extracted Rab to total Rab (extracted + not-extracted) was calculated. Rab10 extracted by empty vector and GDI are shown in *open* and *closed circles*, respectively. Rab29 extracted by empty vector and GDI are shown in *open* and *closed squares*, respectively. The symbols and the error bars represent the mean values and their standard errors, respectively. ∗∗*p* < 0.01, ∗∗∗*p* < 0.001. The *p*-values and statistical tests used are summarized in Table S1. GDI, GDP-dissociation inhibitor.

Rab proteins are thought to exist in the cytosol as a result of their extraction from the membrane by GDIs. Since Rab29 mainly resided in the membrane fraction, we hypothesized that Rab29 cannot be extracted by GDIs. To test this hypothesis, we first conducted an *in vitro* extraction assay using purified GDI1/2 protein. The membrane fraction of HEK293A cells containing endogenous Rab proteins was incubated with recombinant GDI1/2, followed by ultracentrifugation to separate the supernatants containing Rab–GDI complexes (“extracted” fraction) from the precipitates containing the membranes (“not-extracted” fraction). The degree of Rab extraction was quantified as the proportion of Rab in the extracted fraction out of total Rab. In the case of typical Rab proteins which do get extracted by GDIs, adding more recombinant GDI would increase the amount of Rab in the extracted fraction. Indeed, endogenous Rab10 was extracted from the membrane in GDI1- and GDI2-dependent manners (Fig. 2). On the other hand, endogenous Rab29 was not extracted by GDIs (Fig. 2).

**Figure 3:**
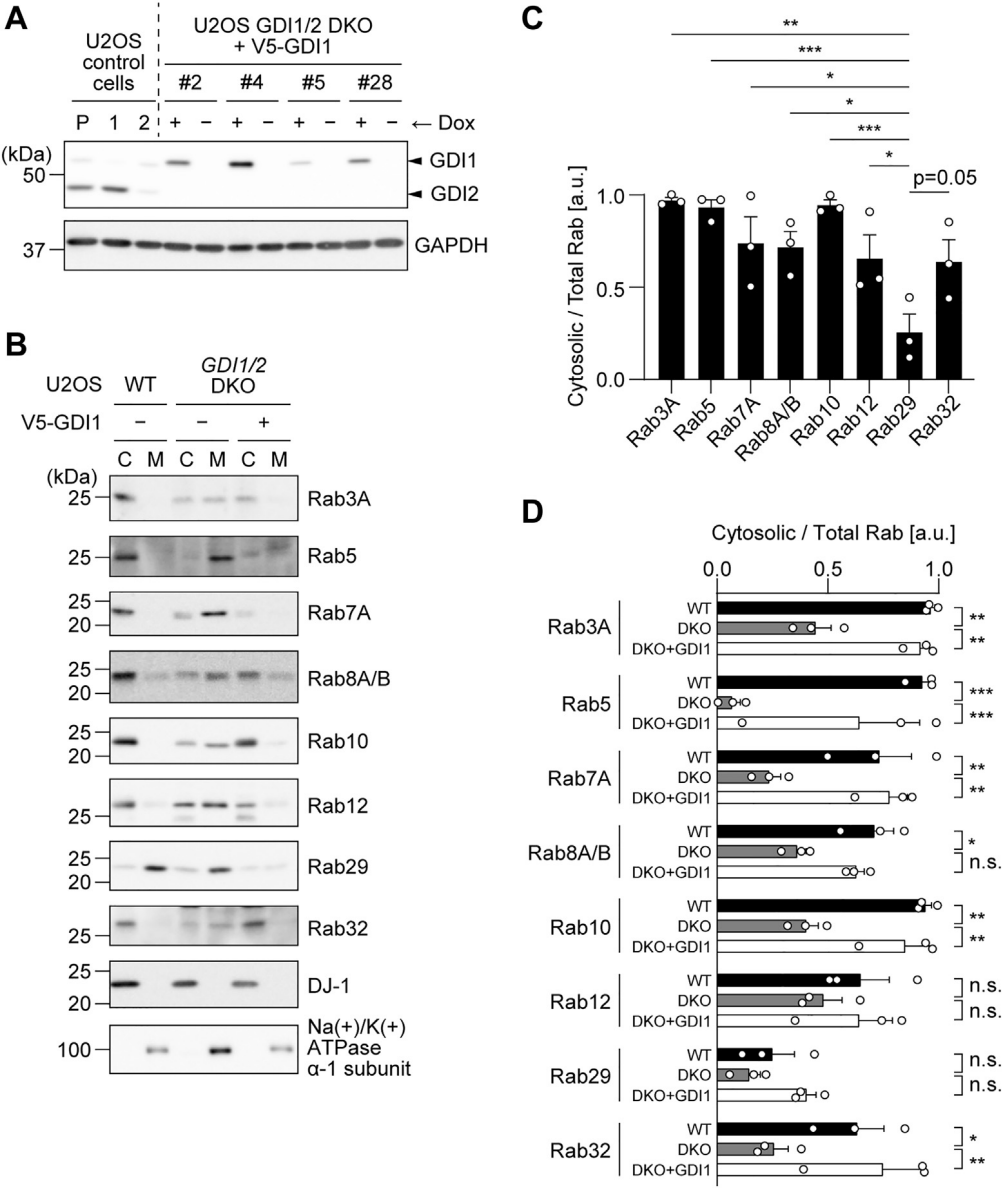
**The effect of GDI1/2 knockout on the localization of Rab proteins.***A*, immunoblots showing the establishment of U2OS clonal cells harboring doxycycline (Dox)-inducible expression of V5-GDI1 in the absence of endogenous GDIs. In the *top panel*, the *upper* and *lower bands* represent GDI1 and GDI2, respectively. The specificity of the anti-GDI antibody used in the *top panel* was examined using parental U2OS cells (P) and U2OS GDI1 KO (1) or GDI2 KO (2) polyclonal cells. Four monoclonal GDI1/2 DKO cells (#2, #4, #5, and #28) were established. GAPDH was used as a loading control (*bottom panel*). *B*, post-nuclear supernatants of parental U2OS cells as well as U2OS GDI1/2 DKO cells treated without (−) or with (+) dox to induce the expression of V5-GDI1 were fractionated into the cytosol (C) and membrane (M) fractions. Representative immunoblots of three independent experiments with the indicated antibodies are shown. DJ-1 was used as a representative cytosolic protein, whereas Na+/K+ ATPase α-1 subunit was used as a membrane protein. *C*, the comparison of the ratio of cytosolic to total Rab proteins among Rab proteins in U2OS cells. *D*, the comparison of the ratio of cytosolic to total Rab proteins between U2OS cells, U2OS GDI1/2 DKO cells without dox (DKO), and U2OS GDI1/2 DKO cells with dox to induce the expression of V5-GDI1 (DKO+GDI1). The band intensity of the Rab proteins was quantified, and the ratio of cytosolic Rab to total Rab (cytosol + membrane) was calculated. The circles, the bars, and the error bars in the graphs represent individual values, the mean values, and their standard errors, respectively. ∗*p* < 0.05, ∗∗*p* < 0.01, ∗∗∗*p* < 0.001. 'n.s.' means 'not significant'. The *p*-values and statistical tests used are summarized in Table S1. DKO, double knockout; GDI, GDP-dissociation inhibitor.

Furthermore, to investigate the regulation by GDIs under the physiological conditions in cells, we performed the knockout of GDIs in U2OS cells. However, simple GDI1/2 double KO (DKO) cells could not be generated, presumably because lacking both GDIs severely impaired cell survival and proliferation (data not shown). Therefore, we first generated U2OS cells that inducibly express V5-GDI1 in the presence of doxycycline (dox). Then, while maintaining the expression of exogenous V5-GDI1, we performed the KO of GDI1 by using a guide RNA (gRNA) pair that targets an intron, thereby specifically knocking out the endogenous GDI1 gene. Simultaneously, GDI2 was knocked out using a gRNA pair that targets an exon. Single cell cloning was conducted, and four clones (#2, #4, #5, and #28) were generated. It was confirmed that dox withdrawal resulted in the transient DKO of GDI1/2 (Fig. 3*A*).

Next, to examine the effect of GDI1/2 DKO on cytosol/membrane localization of Rab proteins, U2OS WT as well as GDI1/2 DKO #5 cells without (−) or with (+) dox were subjected to fractionation by ultracentrifugation, and the ratio of cytosolic to total Rab proteins was examined by immunoblotting (Fig. 3*B*). In WT cells, Rab3A, Rab5, Rab7A, Rab8A/B, Rab10, Rab12, and Rab32 were mainly found in the cytosolic fraction, whereas Rab29 was predominantly fractionated into the membrane fraction as described in Figure 1 (Fig. 3*C*). In GDI1/2 DKO #5 cells without dox, the amount of Rab proteins in the cytosolic fraction was decreased in most of the Rab proteins (Fig. 3, *B* and *D*). The cytosolic localization of these Rab proteins was rescued with the dox-inducible expression of V5-GDI1, indicating that the change in localization in GDI1/2 DKO #5 cells was an on-target effect. On the other hand, Rab29 was found mainly in the membrane fraction in GDI1/2 DKO #5 cells as well as in WT cells, suggesting that while typical Rab proteins exist in the cytosol in a GDI-dependent manner, Rab29 exists in the membrane in a manner that is not affected by the presence of GDIs.

**Figure 4:**
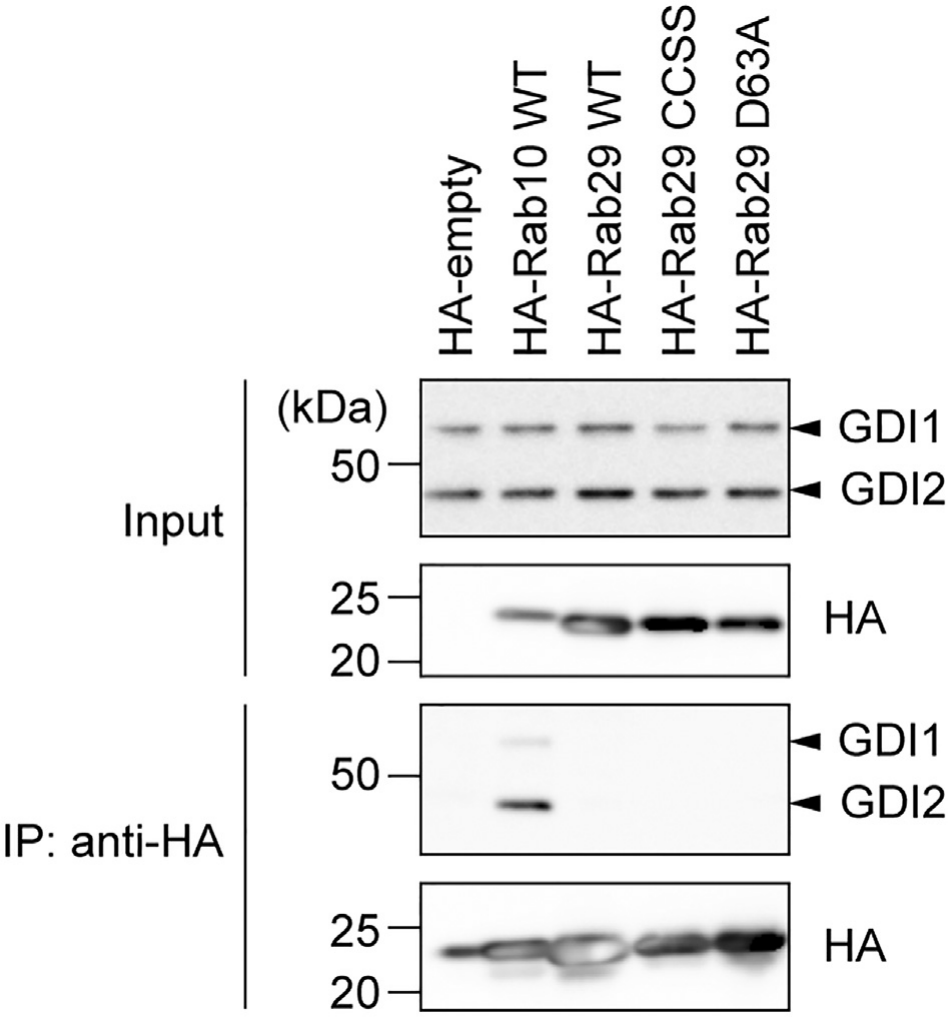
**Coimmunoprecipitation of GDI1/2 with Rab proteins.** HA-empty, HA-Rab10, HA-Rab29 WT, HA-Rab29 C202S+C203S (CCSS), or HA-Rab29 D63A were overexpressed in HEK293A cells and immunoprecipitated using HA-agarose beads (the *bottom panel*). Coimmunoprecipitation of GDI1 and GDI2 with the overexpressed HA-tagged proteins was examined by immunoblotting (the *second panel* from the *bottom*). Equal levels of the expression of GDI1/2 as well as HA-tagged proteins were shown in the *top panels* (input). GDI, GDP-dissociation inhibitor.

We also examined the subcellular localization of Rab10 and Rab29 in GDI1/2 DKO #5 cells by immunocytochemistry (Fig. S2). In the absence of dox, HA-Rab10 stably overexpressed in GDI1/2 DKO #5 cells appeared to form large aggregates in the perinuclear region with some cytosolic diffuse staining, whereas, in the presence of dox, vesicular structures presumably corresponding to active Rab10 on vesicles became evident. In contrast, HA-Rab29 stably overexpressed in GDI1/2 DKO #5 cells appeared to be punctate or vesicular in the perinuclear region and reticular in the cytosol regardless of the presence or absence of dox. This might correspond to the membranes of the Golgi and the endoplasmic reticulum (ER), respectively. These results are consistent with the biochemical fractionation by ultracentrifugation. We also investigated the interaction of Rab29 and GDIs in cells. HEK293A cells were transiently overexpressed with either HA-Rab10 (WT) or HA-Rab29 (WT), and immunoprecipitation by an HA antibody was performed. GDI1/2 was coimmunoprecipitated with HA-Rab10 but not with HA-Rab29 (Fig. 4).

Taken together, these results show that Rab29 is not extracted from the membrane by GDIs not only *in vitro* but also under the physiological condition of cells.

### Rab29 is hydrophobic to a similar extent as other Rab proteins

In considering why Rab29 is not regulated by GDIs, we focused on the geranylgeranylation which typically occurs at the carboxyl terminus of Rab proteins. It has been shown that geranylgeranylation of Rab proteins significantly enhances their binding to, and extraction by, GDIs (23). Therefore, we hypothesized that Rab29 may be deficient in binding to GDIs due to the lack of geranylgeranylation.

**Figure 5:**
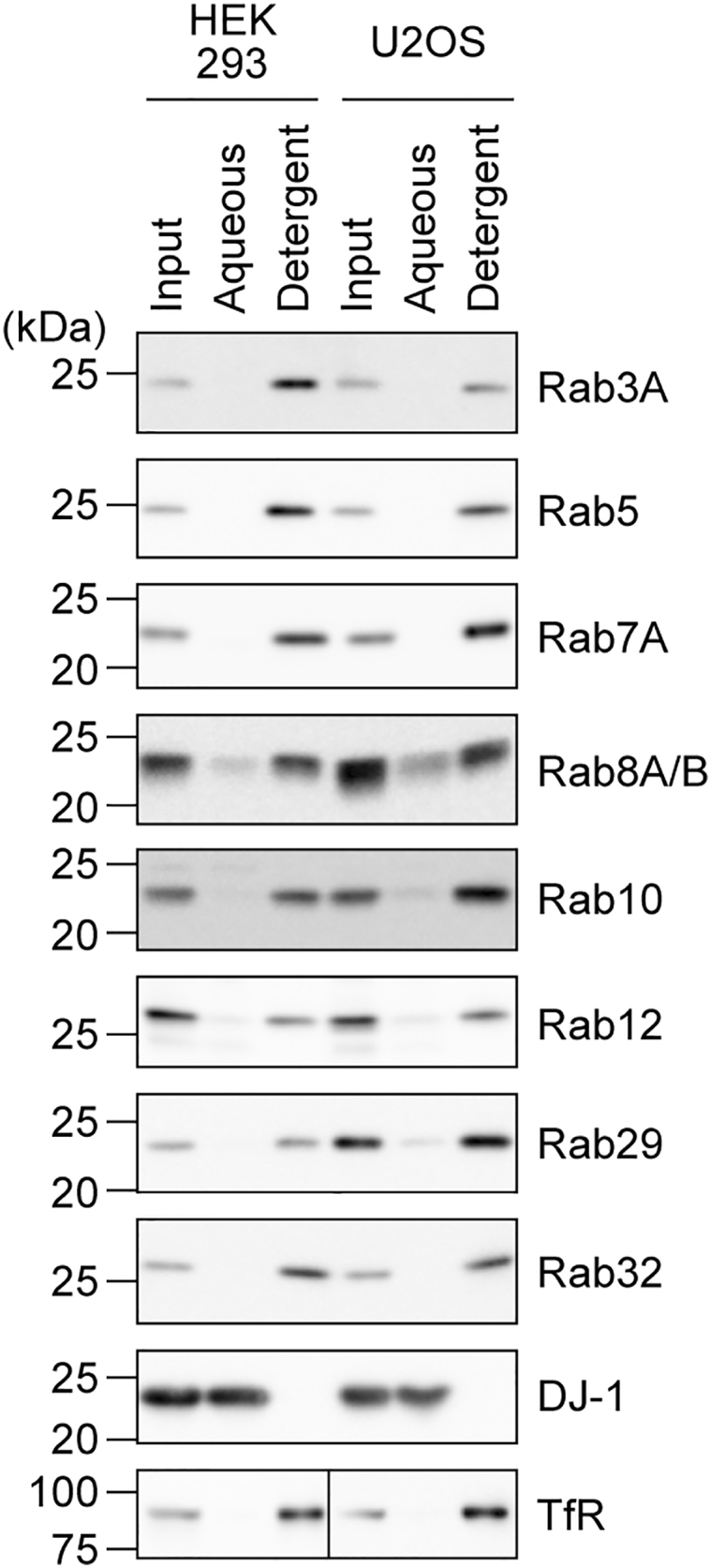
**Fractionation of various Rab proteins depending on their hydrophobicity using Triton X-114.** HEK293A and U2OS cells were lysed in a buffer containing Triton X-114. Cleared lysates (input) were heated to 37 °C to cause phase separation of Triton X-114. The aqueous and detergent phases were subjected to immunoblotting with the indicated antibodies. DJ-1 was used as a representative hydrophilic protein, whereas the transferrin receptor was used as a hydrophobic protein.

Geranylgeranylation is the most hydrophobic modification among prenylation. Conjugation of one or two geranylgeranyl moieties on the carboxyl-terminal cysteine(s) greatly increases the hydrophobicity of the modified Rab proteins. Lysing cells with Triton X-114 (TX114) on ice and subsequently warming the cleared lysate up to 37 °C leads to the phase separation of TX114, resulting in the aqueous phase and the TX114 (detergent) phase. Here, in general, hydrophilic proteins are found in the aqueous phase, whereas proteins with hydrophobic modifications such as geranylgeranylation are found in the detergent phase (24). We utilized this method to investigate the geranylgeranylation of Rab proteins. HEK293A cells or U2OS cells were subjected to phase separation using TX114. The validity of this separation was confirmed from the result showing that a hydrophilic protein DJ-1 was fractionated into the aqueous phase, while the transferrin receptor, which is a transmembrane protein modified by palmitoylation and is therefore hydrophobic, was fractionated into the detergent phase (25) (Fig. 5). Rab29 was mostly fractionated into the detergent phase, as were the cases with other Rab proteins (Fig. 5). These results indicated that endogenous Rab29 is modified by a highly hydrophobic lipid modification.

**Figure 6:**
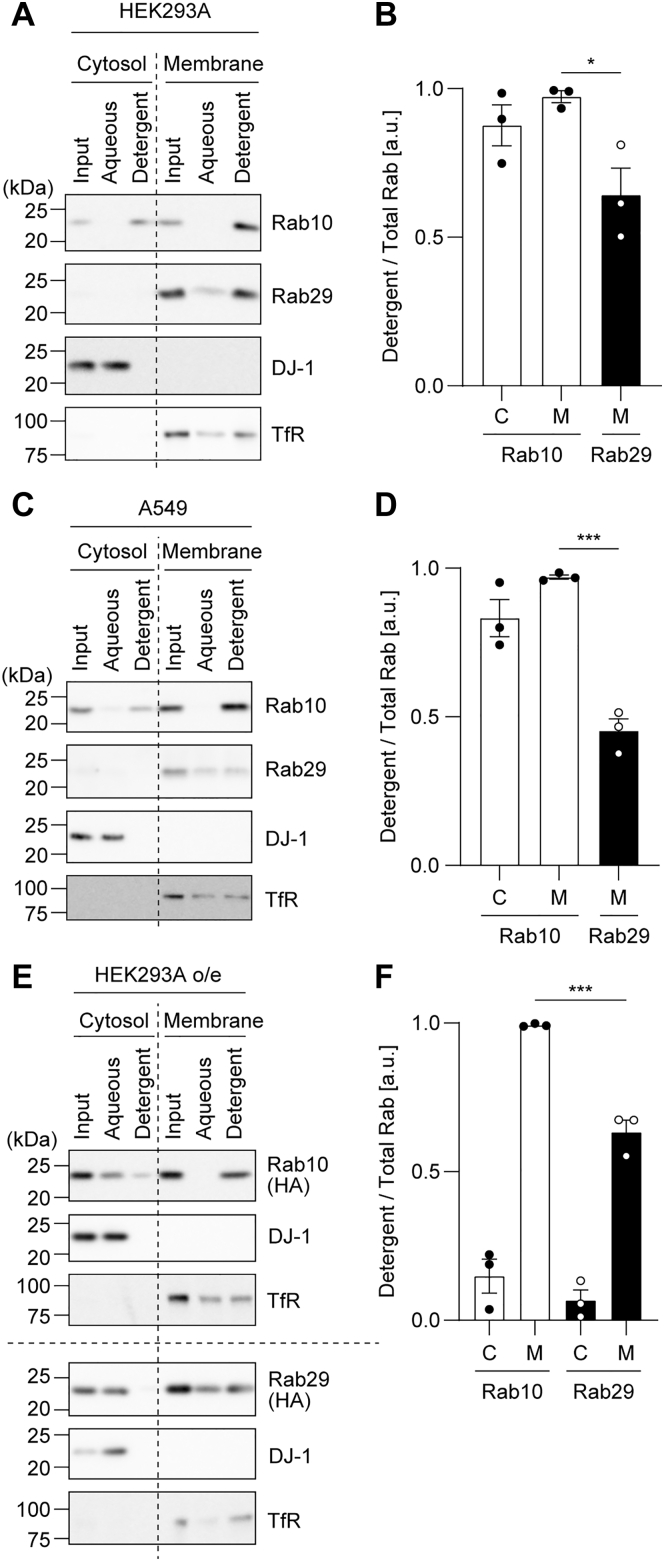
**Fractionation of Rab proteins in the cytosol and membrane fractions using Triton X-114.** Post-nuclear supernatants of HEK293A (*A* and *B*), A549 (*C* and *D*), and HEK293A cells overexpressing HA-Rab10 or HA-Rab29 (*E* and *F*) were fractionated into the cytosol and membrane fractions by ultracentrifugation. Each fraction was further fractionated using Triton X-114 into aqueous and detergent phases and subjected to immunoblotting with the indicated antibodies. DJ-1 was used as a representative hydrophilic protein, whereas the transferrin receptor was used as a hydrophobic protein. Note that, for this experiment, equal amounts of protein, not volumes of the samples, were loaded for the cytosol and membrane fractions. The amount of Rab10 in the membrane fraction seems larger than that in the cytosol fraction, but this does not reflect the actual ratio of the amounts of Rab10 in the cytosol and membrane fractions as shown in Figure 1. Also, note that the transferrin receptor was observed both in the aqueous and detergent phases only when the cells were first mechanically homogenized for the fractionation by ultracentrifugation (*A*, *C*, and *E*). The band intensity of the Rab proteins was quantified, and the ratio of Rab in the detergent phase to total Rab (aqueous + detergent) was calculated. The circles, the bars, and the error bars in the graphs represent individual values, the mean values, and their standard errors, respectively. ∗*p* < 0.05, ∗∗∗*p* < 0.001. The *p*-values and statistical tests used are summarized in Table S1.

Next, HEK293A cells were fractionated by ultracentrifugation, and the resulting membrane fraction and cytosol fraction were separately subjected to TX114 phase separation. Both Rab10 in the cytosol fraction and Rab10 in the membrane fraction were found in the detergent phase (Fig. 6, *A* and *B*). This result indicated that Rab10 in the cytosol fraction is as hydrophobic as that in the membrane fraction, presumably existing as a complex with GDIs. On the other hand, endogenous Rab29 resided mainly in the membrane fraction, and approximately 60% of this was fractionated into the detergent phase, while about 40% was found in the aqueous phase (Fig. 6, *A* and *B*). From this result, it was suggested that the majority of membrane-bound Rab29 is hydrophobic due to lipid modification, while there is a minor pool of hydrophilic Rab29 in the membrane fraction. Similar results were obtained with A549 cells (Fig. 6, *C* and *D*), where we found that approximately 60% of Rab29 in the membrane fraction is hydrophilic.

We then overexpressed HA-Rab10 or HA-Rab29 in HEK293A cells and subjected the cells to fractionation by ultracentrifugation, followed by TX114 phase separation of each fraction. As for Rab10 and Rab29 in the membrane fraction, the fractionation pattern was similar to that of endogenous Rab10 and Rab29, with Rab10 almost completely being fractionated into the detergent phase, while a small part of Rab29 was found in the aqueous phase (Fig. 6, *E* and *F*). On the other hand, overexpressed Rab10 and Rab29 in the cytosol fraction showed a fractionation pattern different from that of endogenous ones. Specifically, overexpressed Rab10 in the cytosol was mainly found in the aqueous phase (Fig. 6, *E* and *F*). Also, when overexpressed, Rab29 is partially fractionated into the cytosol, and this was almost entirely fractionated into the aqueous phase (Fig. 6, *E* and *F*). As transient overexpression of proteins in cultured cells tends to produce immature proteins, we speculate that, upon overexpression, a larger portion of the Rab proteins lacking lipid modification came to exist in the cytosol fraction, leading to the increased hydrophilic rate in the TX114 phase separation.

### Evidence of geranylgeranylation of Rab29

It has been well described that Rab proteins are geranylgeranylated on one or two cysteine residue(s), which is a prerequisite for their membrane localization. We further set out to investigate whether the lipid modification conferring hydrophobicity on Rab29 is geranylgeranylation as is with other Rab proteins. This was achieved by several methods.

**Figure 7:**
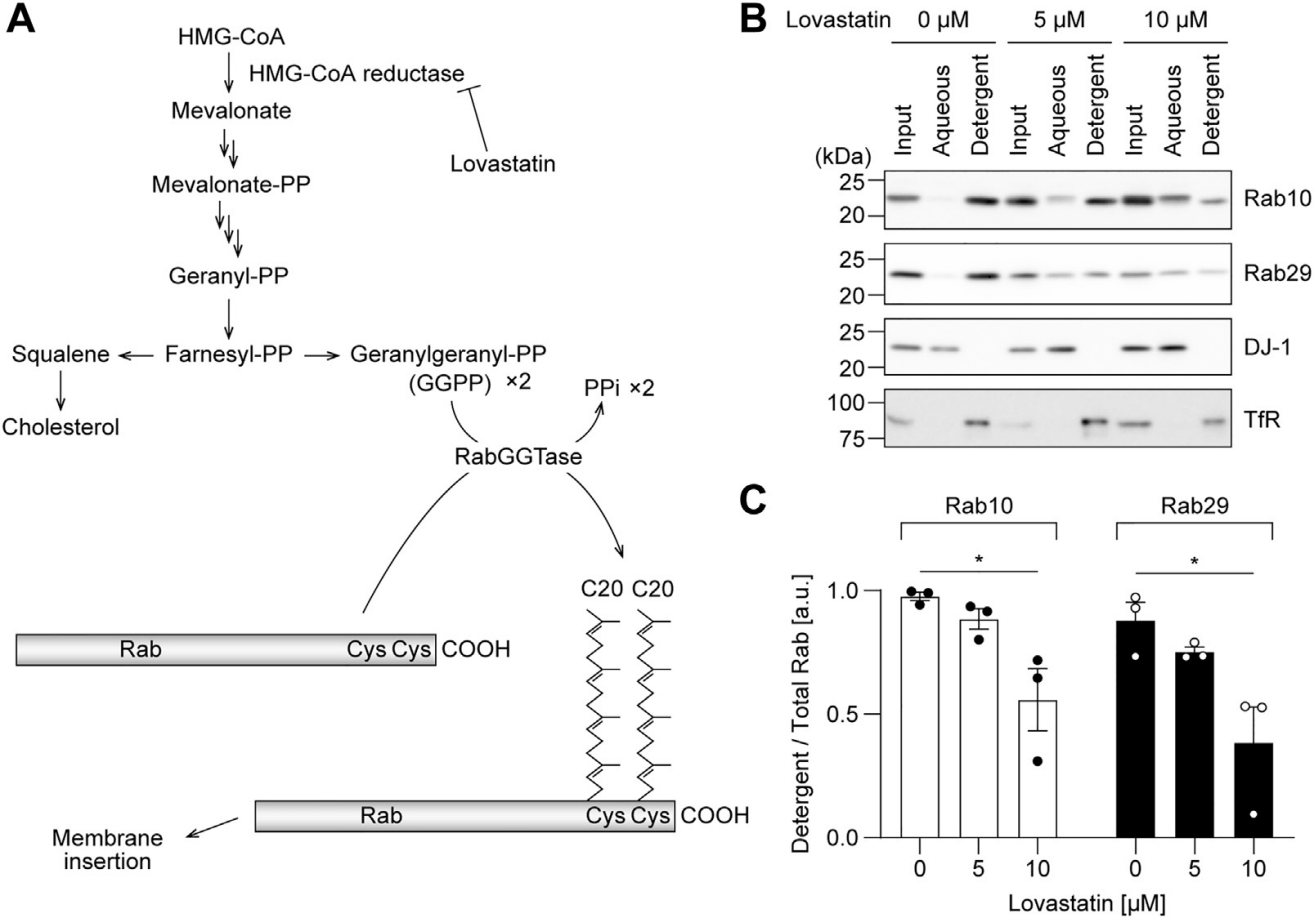
**The effect of lovastatin treatment on the hydrophobicity of Rab10 and Rab29.** HEK293A cells were treated with the indicated concentration of lovastatin for 36 h and fractionated using Triton X-114. *A*, a schematic illustration of how lovastatin treatment inhibits geranylgeranylation of Rab proteins. *B*, representative immunoblots of three independent experiments with the indicated antibodies are shown. DJ-1 was used as a representative hydrophilic protein, whereas the transferrin receptor was used as a hydrophobic protein. *C*, the band intensity of the Rab proteins was quantified, and the ratio of Rab in the detergent phase to total Rab (aqueous + detergent) was calculated. The circles, the bars, and the error bars in the graphs represent individual values, the mean values, and their standard errors, respectively. ∗*p* < 0.05. The *p*-values and statistical tests used are summarized in Table S1.

The geranylgeranylation of Rab proteins is catalyzed by an enzyme complex, RabGGTase, which transfers the geranylgeranyl moiety from geranylgeranyl diphosphate (GGPP) to the thiol group of a cysteine residue. GGPP is synthesized from mevalonate, which is an intermediate of the cholesterol biosynthesis pathway, through the synthesis of geranyl diphosphate and farnesyl diphosphate (Fig. 7*A*). Therefore, treating cells with lovastatin, an inhibitor of 3-hydroxy-3-methyl-glutaryl-coenzyme A reductase, which synthesizes mevalonate, results in the depletion of GGPP and farnesyl diphosphate, leading to the inhibition of geranylgeranylation and farnesylation. To investigate what modification is conferring hydrophobicity to Rab29 in cells, HEK293A cells were treated with lovastatin and then subjected to TX114 phase separation. With increasing amounts of lovastatin, both Rab10 and Rab29 were fractionated less in the detergent phase and more in the aqueous phase (Fig. 7, *B* and *C*). These results indicated that Rab29 is modified either by geranylgeranylation or by farnesylation.

**Figure 8:**
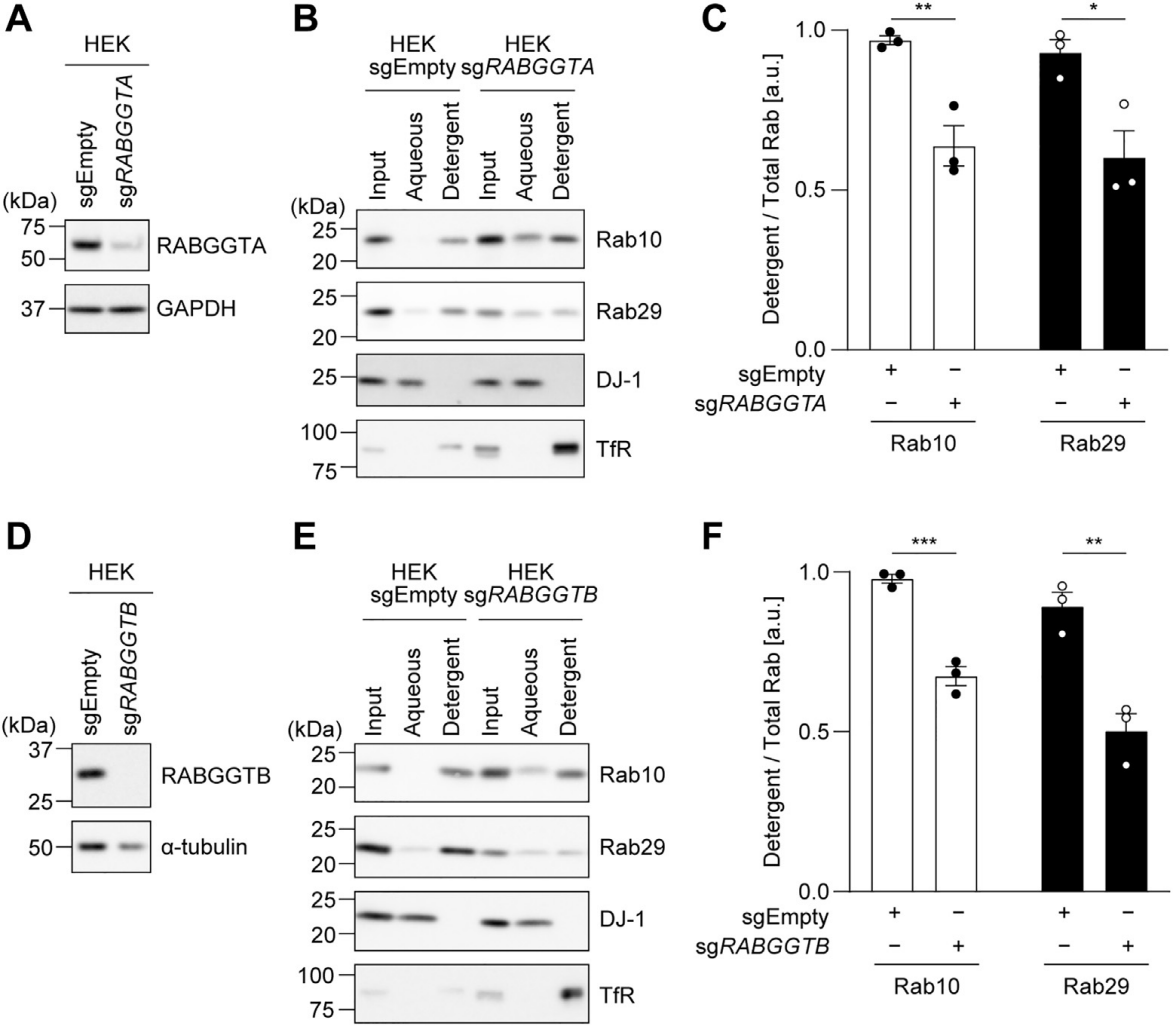
**The effect of the knockout of RABGGTA and RABGGTB on the hydrophobicity of Rab10 and Rab29.** HEK293A cells lacking *RABGGTA* or *RABGGTB* gene were fractionated using Triton X-114 (*A*–*C*, *RABGGTA* KO cells; *D*–*F*, *RABGGTB* KO cells). *A*, *B*, *D*, and *E*, representative immunoblots of three independent experiments with the indicated antibodies are shown. DJ-1 was used as a representative hydrophilic protein, whereas the transferrin receptor was used as a hydrophobic protein. *C* and *F*, the band intensities of the Rab proteins were quantified, and the ratio of Rab in the detergent phase to total Rab (aqueous + detergent) was calculated. The circles, the bars, and the error bars in the graphs represent individual values, the mean values, and their standard errors, respectively. ∗*p* < 0.05, ∗∗*p* < 0.01, ∗∗∗*p* < 0.001. The *p*-values and statistical tests used are summarized in Table S1.

After translation, REPs recognize the immature Rab proteins and recruit them to the RabGGTase complex. RabGGTase consists of the α subunit (RABGGTA) responsible for binding REPs and the β subunit (RABGGTB), which contains the catalytic domain. Therefore, to elucidate whether Rab29 is geranylgeranylated, we performed the knockout of each of these subunits. RABGGTA/B was knocked out in HEK293A cells (Fig. 8, *A* and *D*), and these cells in the polyclonal state were subjected to TX114 phase separation. Upon knockout of either gene, the hydrophobicity of both Rab10 and Rab29 decreased (Fig. 8, *B*, *C*, *E* and *F*). This result indicated that, like other Rab proteins, Rab29 is modified by geranylgeranylation by RabGGTase. In both KO cells, the amount of Rab29 in the aqueous phase did not seem to increase, but instead, the amount in the detergent phase seemed to decrease (Fig. 8, *B* and *E*). This result hints at the possibility that immature Rab29, which is not geranylgeranylated, is unstable and is more readily degraded than mature Rab29.

### D63A mutation reduces the membrane localization and the hydrophobicity of Rab29

**Figure 9:**
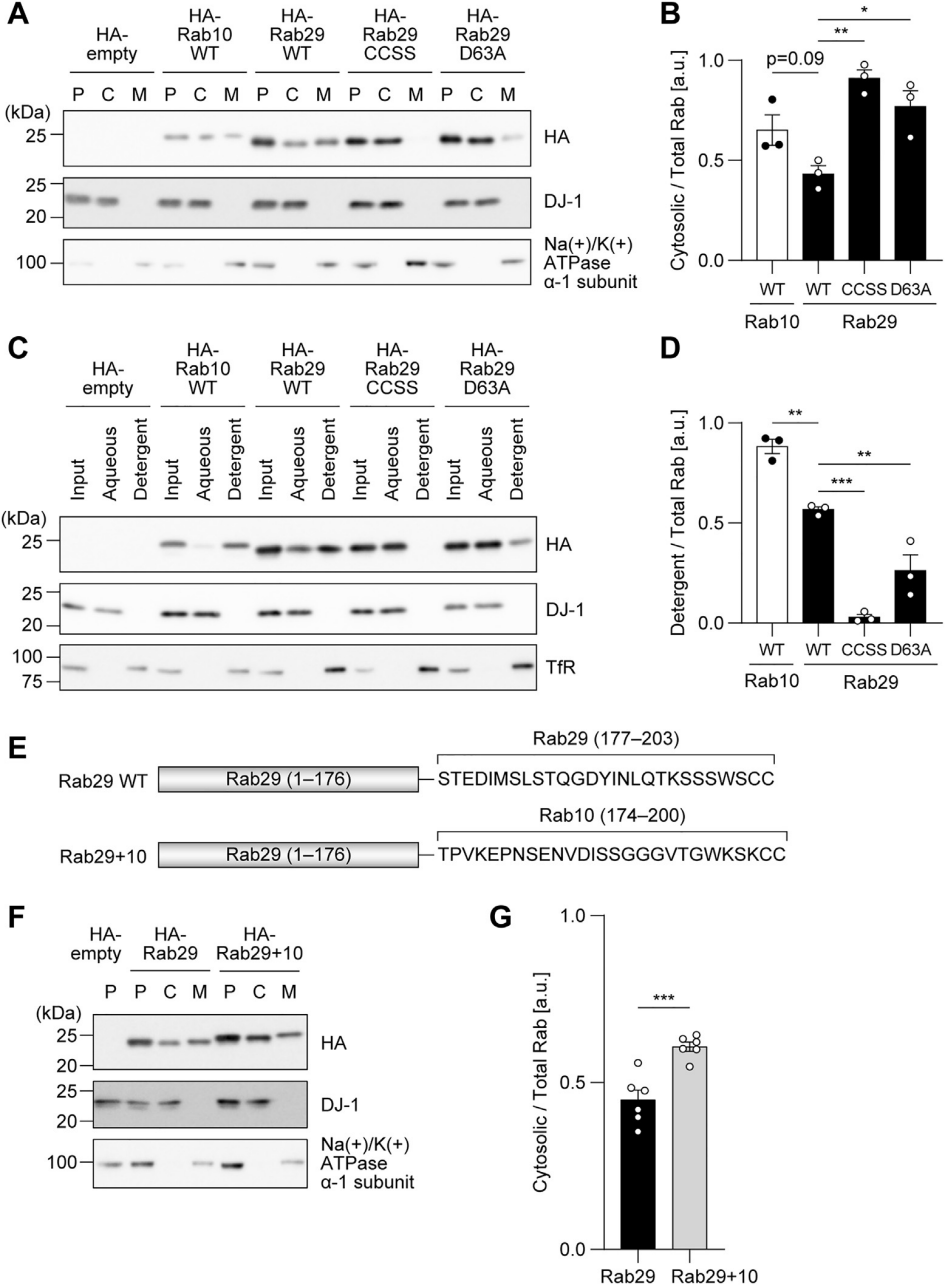
**The effect of the D63A mutation and the carboxyl-terminal domain swap on the membrane localization and the hydrophobicity of Rab29.***A*, post-nuclear supernatants (P) of HEK293A cells overexpressing HA-empty, HA-Rab10 WT, HA-Rab29 WT, HA-Rab29 CCSS, or HA-Rab29 D63A were fractionated into the cytosol (C) and membrane (M) fractions by ultracentrifugation. Representative immunoblots of three independent experiments with the indicated antibodies are shown. DJ-1 was used as a representative cytosolic protein, whereas Na+/K+ ATPase α-1 subunit was used as a membrane protein. *B*, the band intensity of the Rab proteins in (*A*) was quantified, and the ratio of cytosolic Rab to total Rab (cytosol + membrane) was calculated. *C*, HEK293 cells overexpressing HA-empty, HA-Rab10 WT, HA-Rab29 WT, HA-Rab29 CCSS, or HA-Rab29 D63A were fractionated using Triton X-114 into aqueous and detergent phases. Representative immunoblots of three independent experiments with the indicated antibodies are shown. DJ-1 was used as a representative hydrophilic protein, whereas the transferrin receptor was used as a hydrophobic protein. *D*, the band intensity of the Rab proteins was quantified, and the ratio of Rab in the detergent phase to total Rab (aqueous + detergent) was calculated. *E*, an illustration of a Rab29 chimeric protein (Rab29+10) in which the carboxyl-terminal sequence (177–203 aa) was swapped with that of Rab10 (174–200 aa) in comparison with Rab29 WT. *F*, post-nuclear supernatants (P) of HEK293A cells overexpressing HA-Rab29 WT or HA-Rab29+10 were fractionated into the cytosol (C) and membrane (M) fractions by ultracentrifugation. Representative immunoblots of six replicates with the indicated antibodies are shown. Similar results were obtained in six independent experiments. DJ-1 was used as a representative cytosolic protein, whereas Na+/K+ ATPase α-1 subunit was used as a membrane protein. *G*, the band intensities of the Rab proteins in (*F*) was quantified, and the ratio of cytosolic Rab to total Rab (cytosol + membrane) was calculated. The circles, the bars, and the error bars in the graphs represent individual values, the mean values, and their standard errors (*B* and *D*) or their standard deviations (*G*), respectively. ∗*p* < 0.05, ∗∗*p* < 0.01, ∗∗∗*p* < 0.001. The *p*-values and statistical tests used are summarized in Table S1.

Next, to investigate the relationship between the nucleotide-binding state of Rab29 and its localization, we utilized the D63A mutant of Rab29 (D63A Rab29). This mutant was reported to release the bound nucleotide very rapidly *in vitro*, presumably due to its low ability to bind the magnesium ion and is predicted to be nucleotide-free in cells as well (22). When D63A Rab29 was overexpressed in HEK293A cells, it was mainly detected in the cytosol fraction after ultracentrifugation fractionation (Fig. 9, *A* and *B*). In addition, TX114 phase separation demonstrated that this mutant was more hydrophilic than WT Rab29, suggesting the low efficiency of the geranylgeranylation of this mutant (Fig. 9, *C* and *D*). This was in line with the result showing that D63A Rab29 did not bind to GDI1/2, as was the case with WT Rab29 (Fig. 4). Therefore, it can be predicted that the D63A mutant exists in the cytosol not as a result of extraction by GDI through its geranylgeranylation motif but instead in an immature form that is not geranylgeranylated.

Furthermore, the same analysis was performed with the CCSS mutant of Rab29 (CCSS Rab29), in which Cys202 and Cys203 at the carboxyl terminus, the predicted geranylgeranylation sites, are both substituted with serines. As expected, the CCSS Rab29 almost completely existed in the cytosol, presumably due to the lack of geranylgeranylation (Fig. 9, *A* and *B*). As for the TX114 phase separation, this mutant was found in the aqueous phase (Fig. 9, *C* and *D*). Therefore, it was suggested that Rab29 resides in the membrane as a result of geranylgeranylation at the cysteine(s) at its carboxyl terminus.

## Discussion

In the present study, we systematically characterized the membrane localization of Rab29 in comparison with other Rab proteins, especially Rab10, using biochemical fractionation by ultracentrifugation as well as phase separation using Triton X-114. We found that in all types of cells and tissues examined, Rab29 was fractionated predominantly in the membrane fraction and was not affected by the knockout of GDI1/2, while other Rab proteins were mainly fractionated in the cytosol fraction and decreased its cytosolic localization by the knockout of GDI1/2.

Of note, Rab12 was the exception among other Rab proteins in that it was not significantly influenced by the knockout of GDI1/2, with a slight but not significant decrease of cytosolic Rab12 in GDI1/2 DKO cells compared with WT cells (Fig. 3*D*). A possible cause for this might be that the cytosolic localization of Rab12 is partly regulated by proteins other than GDIs. There are a few known examples of such GDI-independent regulation for other Rab proteins: chroideremia-like (also known as REP2) has GDI-like roles in binding GDP-bound Rab14 and escorting it to the membrane (26), and BAG6 binds GDP-bound Rab8A to stabilize it in the cytosol (27). In this way, factors other than GDIs may have influenced the localization of Rab12.

Rab29 is fractionated into the membrane fraction, presumably due to its hydrophobicity conferred by the geranylgeranylation at the carboxyl-terminal cysteine residues. Although our efforts on directly identifying the nature of the lipid modification were in vain, our results showing that the lovastatin treatment of cells and the knockout of RabGGTase subunits decreased the hydrophobicity of Rab29 strongly indicate that the modification conferring hydrophobicity on Rab29 is geranylgeranylation. Intriguingly, 40 to 60% of Rab29 in the membrane fraction was found in the aqueous phase in the TX114 phase separation (Fig. 6, *A*–*D*). This points to the possibility that the modification of Rab29 may not be limited to the two geranylgeranylations at the two cysteines at their carboxyl terminus as commonly perceived for other Rabs with these residues. Another modification pattern may be possible such that a more hydrophilic type of Rab29 exists on the membrane. Alternatively, there may be a pool of Rab29 that is associated with the membrane independently of its lipid modification. However, this is in contradiction with the result showing that the CCSS mutant, which is deficient in geranylgeranylation, was almost completely fractionated into the cytosol (Fig. 9, *A* and *B*).

As reported by Gomez *et al.* (22), Rab29 overexpressed in HEK293A cells failed to bind to GDIs (Fig. 4). Although overexpression of Rab29 increased the pool of immature Rab29 devoid of geranylgeranylation (Fig. 6*E*), this should not be the cause of the failure of coimmunoprecipitation of GDIs with Rab29, as Rab10 overexpressed similarly was able to coimmunoprecipitate GDIs (Fig. 4).

Several causes can be suggested for the deficiency of Rab29 in being extracted by GDI1/2. One is that Rab29 lacks the amino acids important for GDI binding or that its sequence weakens its binding to GDIs. Indeed, when the carboxyl-terminal sequence of Rab29 was replaced with that of Rab10, the amount of the chimeric Rab29 in the cytosol fraction was slightly but significantly increased, suggesting that the carboxyl-terminal region of Rab29 plays a role in its membrane localization (Fig. 9, *E*–*G*). Of note, Rab32 that belongs to the same subfamily as Rab29 along with Rab38, resided mainly in the cytosol fraction, unlike Rab29, and its membrane localization was significantly increased in GDI1/2 DKO cells (Fig. 3*B*). These results suggest that Rab29 has a unique property even within the subfamily.

A second possible cause for the deficiency of Rab29 in being extracted by GDI1/2 is that Rab29 interacts with proteins which preclude the extraction of Rab29 from the membrane by GDIs. It has been reported that a protein named RABAC1/PRAF1 inhibits the extraction of Rab3A by GDIs (28). RABAC1/PRAF1 is one of the PRAF (prenylated Rab acceptor 1 domain family) proteins, and other members include PRAF2 and ARL6IP5/PRAF3. The knockdown of each of these three family members was performed to investigate the involvement of these proteins in the membrane localization of Rab29. However, the knockdown of these proteins by siRNA did not change the membrane localization of Rab29 (Fig. S3). Similarly, it is also possible that the lipid composition of the specific membrane compartment where Rab29 is localized interferes with its efficient extraction from the membrane by GDIs. Indeed, it has been shown that the extraction of Rab7A by GDI becomes less efficient when the amount of cholesterol in the membrane is increased (29). A number of reports have shown that Rab29 is specifically localized to the *trans*-Golgi network (7, 9, 11, 30, 31, 32, 33). Furthermore, in our experiments, WT Rab29 seemed to be localized to the ER in addition to the Golgi (Fig. S4). Therefore, future studies will be required to investigate the effects of the interacting partners of Rab29 on the *trans*-Golgi network and on the ER and the lipid composition of these membranes on the extraction of Rab29 by GDIs.

Thirdly, it is also possible that, unlike other Rab proteins, most of Rab29 exists in the GTP-bound form, which cannot be extracted by GDI1/2, leading to its preferential localization on the membrane. This possibility needs to be investigated in the future; for example, by using a pull-down assay with a Rab-binding domain of yet-to-be-identified Rab29 effector proteins. On the contrary, some previous reports have shown that GDP-bound Rab29 is partially localized to the Golgi membranes (11, 33), although in our experiments, Rab29 harboring a mutation (T21N) that generally mimics the GDP-bound form was distributed diffusely (Fig. S4 and Table S2). Therefore, it remains possible that Rab29 can cycle between GTP-bound and GDP-bound forms without extraction by GDIs. In this case, the vesicular transport that Rab29 functions as a molecular switch might not be between spatially distinct membranes.

In summary, we found that Rab29 undergoes geranylgeranylation but fails to be extracted by GDIs, resulting in the predominant localization in the membrane, which is a feature not observed with other Rab proteins. Further studies on the regulation of the activity and localization of Rab29, including how Rab29 becomes specifically localized to the Golgi and/or ER membranes, will provide clues for the elucidation of the physiological functions of Rab29 and its involvement in PD pathogenesis.

## Experimental procedures

### Animal experiments

All experiments using animals in this study were approved by the Institutional Animal Care Committee of the Graduate School of Pharmaceutical Sciences at the University of Tokyo and were performed according to the guidelines provided by the Committee.

### Antibodies

The details of antibodies used in this study are listed in Table S3.

### cDNA cloning and plasmid construction

A plasmid for bacterial expression encoding an amino-terminally 6His-SUMO1-tagged WT human Rab29 (hRab29/pET-15b-6His-SUMO1; DU50291) and a plasmid for dox-inducible expression in mammalian cells encoding amino-terminally HA-tagged WT human Rab10 (HA-hRab10/pcDNA5D FRT TO; DU44250) were kindly provided by Professor Dario Alessi (University of Dundee) and described previously (34). Complementary DNA (cDNA) libraries were prepared from human brain total RNA (TaKaRa Bio Inc) using a PrimeScript II 1st strand cDNA synthesis kit with the oligo dT primer (TaKaRa Bio Inc) according to the manufacturer’s instructions. For plasmid construction, KOD -plus- neo-DNA polymerase (Toyobo) was used for PCR. All oligonucleotides were synthesized by Eurofins Genomics. Generated plasmids were sequence-verified by Sanger sequencing (Eurofins Genomics).

The cDNAs encoding human GDI1 (GI#A092) and GDI2 (GI#A495) were amplified from the human brain cDNA library by PCR. Primers used for cloning are listed in Table S4. The amplified fragments were subjected to 3′-A addition using Gene Taq DNA polymerase (Nippon Gene) and inserted into pCR2.1-TOPO or pCR4-TOPO by TOPO-TA cloning according to the manufacturer’s instructions (Thermo Fisher Scientific).

The following empty vectors were generated and used for subcloning: pET-15b-6His-SUMO1 (GI#A470) for bacterial expression of amino-terminally 6His-SUMO1-tagged proteins, pcDNA5G FRT TO HA-N (GI#A088) for overexpression of amino-terminally HA-tagged protein in mammalian cells, pcDNA3.1 Puro (GI#B114) for overexpression of proteins in mammalian cells, pX459dual D10A (GI#A738) for expression of two guide RNAs, and Cas9 D10A nickase for the CRISPR/Cas9 system as well as for the expression of the puromycin-resistant gene. The following constructs were generated and used for bacterial protein expression: hGDI1 WT/pET-15b-6His-SUMO1 (GI#A520) and hGDI2 WT/pET-15b-6His-SUMO1 (GI#A521). The following constructs were generated and used for overexpression in mammalian cells: V5-hGDI1 WT/pcDNA5G FRT TO (GI#A515), HA-hRab29 WT/pcDNA5G FRT TO (GI#A077), HA-hRab29 C202S+C203S (CCSS)/pcDNA5G FRT TO (GI#B029), HA-hRab29 Q67L/pcDNA5G FRT TO (GI#A620), HA-hRab29 T21N/pcDNA5G FRT TO (GI#A619), HA-hRab29 D63A/pcDNA5G FRT TO (GI#B027), HA-hRab10 WT/pcDNA3.1 Puro (GI#A656), and HA-hRab29 WT/pcDNA3.1 Puro (GI#B247). The following constructs were generated and used for CRISPR/Cas9 knockout in mammalian cells: hGDI1Ex6L+R/pX459dual D10A (GI#A831), hGDI2Ex5L+R/pX459dual D10A (GI#A848), hRABGGTAEx2L+R/pX459dual D10A (GI#B080), hRABGGTAEx3L+R/pX459dual D10A (GI#B081), hRABGGTAEx4L+R/pX459dual D10A (GI#B082), hRABGGTBEx1L+R/pX459dual D10A (GI#A996), hRABGGTBEx6L+R/pX459dual D10A (GI#A997), and hRABGGTBEx8L+R/pX459dual D10A (GI#A998). Nucleotide sequences of the target regions are listed in Table S5. All plasmids used in this study and their full details are available from the corresponding author.

### Cell culture and treatment

A549 cells and HEK293A cells were cultured in high-glucose Dulbecco’s modified Eagle’s medium (DMEM; FUJIFILM Wako Chemicals; #044-29765) supplemented with 10% (v/v) fetal bovine serum (Biosera), 50 units/ml penicillin, and 50 μg/ml streptomycin at 37 °C in a 5% CO_2_ atmosphere. U2OS Flp-In T-REx cells and HEK293 Flp-In T-REx cells (generous gifts from Professor Dario Alessi) were cultured in the same medium containing 100 μg/ml Zeocin (Thermo Fisher Scientific) and 15 μg/ml blasticidin (FUJIFILM Wako Chemicals). All cell lines were routinely checked for *mycoplasma* contamination by PCR.

For stable expression in U2OS and HEK293 Flp-In T-REx cells, cells were seeded into 6-well plates at a density of 5 × 10^5^ cells/well. Expression plasmids were mixed with pOG44 (Thermo Fisher Scientific) at a ratio of 1:9 using FuGENE6 (Promega) or PEI Max (Polysciences, Inc), according to the manufacturer’s instructions. At 48 h after transfection, cells were split at 1:5 into a 10-cm dish and cultured for 24 h. The medium was then replaced with a fresh one containing 100 μg/ml hygromycin (FUJIFILM Wako Chemicals) and 15 μg/ml blasticidin. The medium was changed daily for 1 week and then every other day until resistant colonies became visible.

For lovastatin treatment, HEK293A cells were treated with vehicle (dimethyl sulfoxide) or lovastatin (FUJIFILM Wako Chemicals) (5 μM or 10 μM) for 36 h, after which the cells were subjected to phase separation using Triton X-114.

For cycloheximide treatment, U2OS Flp-In T-REx cells were treated with 100 μg/ml cycloheximide (FUJIFILM Wako Chemicals) for 48 h, after which the cells were homogenized and fractionated by ultracentrifugation. The blockage of protein synthesis by the cycloheximide treatment was confirmed by immunoblotting with an anti-p53 antibody.

### Generation of GDI1/2 DKO cells

For genome editing using the CRISPR/Cas9 technology, we employed the double nicking method using Cas9 nickase to minimize off-target editing (35), in which paired gRNAs are required for editing. The gRNA sequences used are listed in Table S5.

As no DKO cells were obtained by simple simultaneous knockout of both genes, it was suggested that knockout of both GDI1 and GDI2 was deleterious to cells (data not shown). Therefore, we first established cells expressing V5-hGDI1 in a dox-dependent manner and then knocked out both GDI1 and GDI2 in the presence of dox.

For this purpose, U2OS Flp-In T-REx cells were stably transfected with V5-hGDI1 WT/pcDNA5G FRT TO (GI#A515) together with pOG44 using FuGENE6 (Promega) as described previously. After checking the dox-inducible expression of V5-hGDI1, the cells were then subjected to simultaneous knockout of endogenous GDI1 and GDI2. Given that one of the gRNAs for GDI1 (hGDI1Ex6L) targets the border of intron 5 and exon 6 with its PAM in intron 5, it would only target the endogenous *GDI1* locus but not target V5-hGDI1, which lacks introns. U2OS cells expressing V5-hGDI1 were transfected with hGDI1Ex5L+R/pX459dual D10A (GI#A831) and hGDI2Ex5L+R/pX459dual D10A (GI#A848) at a ratio of 1:1 using FuGENE6. To maintain the stable expression of V5-hGDI1, dox was added to the culture medium at 1 μg/ml throughout the process. At 24 h after transfection, the medium was replaced with fresh ones containing puromycin (FUJIFILM Wako Chemicals) at 2.5 μg/ml. The medium was replaced again at 24 h with puromycin. The medium was changed to fresh ones not containing puromycin at 48 h selection, and the cells were grown to confluence.

Single cell cloning was carried out by limiting dilution in the presence of dox, and clones lacking endogenous GDI2 were selected by immunoblotting. Dox was then withdrawn from the medium of the selected clones and the expression of endogenous GDI1 was examined after 1 week of dox withdrawal. Four clones (*e.g.*, #2, #4, #5, and #28) that exhibited no expression of endogenous GDI1 and GDI2 after the dox withdrawal were expanded, and #5 was used as a representative clone (see Fig. 3). GDI1/2 DKO cells were maintained in the presence of dox at 1 μg/ml and subjected to 1 week of dox withdrawal for preparing DKO cells.

### Transient knockdown using the CRISPR/Cas9 technology or by siRNA transfection

Transient knockdown of proteins in cultured cells was carried out either by transfecting plasmids expressing paired gRNAs and Cas9 nickase as described previously or by transfecting siRNAs. To increase the efficiency of knockdown, we simultaneously targeted three distinct regions in the genome using three paired gRNAs when knocking down a gene. Successful knockdown was confirmed by immunoblotting.

The following pools of four siRNAs for a target mRNA (siGENOME) were purchased from Dharmacon (Horizon Discovery): nontargeting #2 (D-001206-14-05), siRABAC1/PRAF1 (M-020136-00-0005), siPRAF2 (M-019671-01-0005), and siARL6IP5/PRAF3 (M-012229-00-0005). For transfection of siRNAs into U2OS cells, cells were cultured in 6-well dishes at a density of 2 × 10^5^ cells/well 24 h before transfection. Five microliters of Lipofectamine RNAiMAX and 30 pmol of siRNA were added separately into 250 μl of Opti-MEM medium. Both solutions were mixed and incubated for 10 min, and the transfection mixture was added to culture dishes containing 2.5 ml of DMEM to give a final concentration of 10 nM siRNA. Cells were collected and used for experiments 72 h after the siRNA transfection.

### Preparation of mouse primary glial cultures

Primary glial cells, mainly comprised of astrocytes and microglia, were obtained as previously described (36). Briefly, we isolated the cerebrum from postnatal day 2 mice of either sex in ice-cold Hanks’ balanced salt solution not containing Ca/Mg (FUJIFILM Wako Chemicals). The cerebrum was suspended in Hanks’ balanced salt solution containing 0.25% trypsin, 0.1 μl/ml deoxyribonuclease (Nippon Gene), 0.8 mM MgSO_4_ (Kanto Chemical Co), and 1.85 mM CaCl_2_ (Kanto Chemical Co) at 37 °C for 15 min. The obtained cell suspension was passed through a 100 μm cell strainer (Falcon) and centrifuged with a culture medium. The cell pellet was resuspended in the culture medium and seeded on cell culture plates. The culture medium was replaced at 4 days *in vitro*. The enrichment of glial cells was confirmed by immunocytochemistry using anti-glial fibrillary acidic protein and anti-Iba1 antibodies (Fig. S5).

### Protein purification of recombinant GDI1/2

Rosetta2(DE3)pLysS competent cells (Merck Millipore) were transformed with a plasmid encoding 6His-SUMO1-empty (GI#A470), 6His-SUMO1-hGDI1 (GI#A520), or 6His-SUMO1-hGDI2 (GI#A521). A single colony was picked up into the lysogeny broth medium containing 100 μg/ml ampicillin and 25 μg/ml chloramphenicol, and the bacterial cultures were shaken overnight at 37 °C. The overnight bacterial cultures were diluted at 1:20 with the lysogeny broth medium containing ampicillin and chloramphenicol, and the cultures were shaken at 37 °C until *A*^600^ reached 0.4. The temperature was then set to 16 °C, and the cultures were supplemented with a final concentration of 0.5 mM IPTG and shaken for another 24 h at 16 °C. The bacterial cultures were centrifuged at 4000*g* for 10 min at 4 °C and resuspended with ice-cold PBS. The bacterial suspensions were centrifuged again at 4000*g* for 10 min at 4 °C, and the obtained bacterial pellets were snap-frozen in liquid nitrogen and stored at −80 °C until use.

The pellets were resuspended in a buffer (buffer A (50 mM Hepes-NaOH pH 8.0, 250 mM NaCl), 1 mM DTT, Complete protease inhibitor cocktail EDTA-free (Roche), 10 mg/ml lysozyme (FUJIFILM Wako Chemicals), 3000 units/l culture Benzonase (Sigma-Aldrich)) and lysed by sonication in ice-cold water. The bacterial lysates were then centrifuged at 12,000*g* for 30 min at 4 °C. The supernatants were supplemented with a final concentration of 10%(v/v) glycerol, 50 μM ATP, and 20 mM imidazole and filtered through an 0.45 μm PES filter (Merck Millipore). The cleared lysates were incubated with 0.5 ml (bed volume) of Ni-NTA agarose (FUJIFILM Wako Chemicals) for 1 h at 4 °C. The beads were washed with 5 ml of the wash buffer (buffer A, 100 μM ATP, 1 mM DTT, and 20 mM imidazole) five times. Then, bound proteins were eluted six times with 0.5 ml of the wash buffer containing 500 mM imidazole. Fractions containing 6His-tagged proteins were pooled and buffer-exchanged to the storage buffer (50 mM Hepes-NaOH pH 7.5, 150 mM NaCl, 10%(v/v) glycerol, and 1 mM DTT) using PD-10 Desalting Columns (Cytiva). The standard protein yield was 10 ∼ 20 mg from 1 l culture for all constructs.

### *In vitro* extraction of Rab proteins from cell membranes using recombinant GDIs

Cells cultured in a 10-cm dish were washed with 5 ml of ice-cold Dulbecco’s PBS (FUJIFILM Wako Chemicals) two times and harvested with 1.5 ml of the homogenization buffer (50 mM Hepes-KOH pH 7.5, 250 mM sucrose, Complete protease inhibitor cocktail EDTA-free, and 1× PhosSTOP (Roche)) using a cell scraper into an ultracentrifuge tube (for a 50.4Ti rotor (Beckman Coulter Inc)). Cells were homogenized using a Polytron homogenizer and centrifuged on an Optima L-90K (Beckman Coulter Inc) at 800*g* for 10 min at 4 °C using the 50.4Ti rotor. The supernatant (postnuclear supernatant) was centrifuged at 200,000*g* for 15 min at 4 °C to pellet membranes. The supernatant (cytosol fraction) was collected, and the pellet was resuspended with 1.5 ml of the homogenization buffer and centrifuged again. The supernatant was discarded, and the pellet (membrane fraction) was resuspended with 500 μl of the Rab extraction (RE) buffer (homogenization buffer supplemented with 50 μM MgCl_2_ and 1 mM DTT). The membrane suspension was passed through a 21-gauge needle ten times and then a 27-gauge needle ten times using a syringe. The protein concentration of the membrane suspension was measured by the Bradford assay and adjusted to 0.5 mg/ml with the RE buffer.

Fifty micrograms of the membrane suspension was aliquoted, and 6His-SUMO1-empty, 6His-SUMO1-hGDI1, or 6His-SUMO1-hGDI2 was added to the aliquots of the membrane suspension at the indicated concentrations. The reaction mixtures were incubated at 30 °C for 30 min with agitation (1000 rpm). After the incubation, the mixtures were centrifuged at 200,000*g* for 10 min at 4 °C. The supernatant (extracted fraction) was mixed with the 2× SDS-PAGE sample buffer (125 mM Tris–HCl pH 6.8, 4%(w/v) SDS, 0.01%(w/v) bromophenol blue, 20%(v/v) glycerol) containing 2%(v/v) β-mercaptoethanol (BME) for subsequent immunoblotting. The pellet was resuspended with 1 ml of the RE buffer and centrifuged again to wash the pellet. The pellet (non-extracted fraction) was then resuspended with the RE buffer so that the volume became equal to that of the extracted fraction and was mixed with the 2× SDS-PAGE sample buffer containing 2%(v/v) BME. The same volumes of the postnuclear supernatant, extracted and non-extracted fractions, were subjected to immunoblotting using anti-Rab10 and anti-Rab29 antibodies.

### Cell fractionation

Fractionation into cytosol and membrane fractions by ultracentrifugation of cell homogenates was carried out as described previously. Proper fractionation of the cytosol and membranes was validated by immunoblotting using antibodies against DJ-1 and Na^+^/K^+^-ATPase α-1 subunit, respectively. The hydrophobicity of proteins was examined by the Triton X-114 phase fractionation as described previously (24). Briefly, cells cultured in the 10 cm dish were washed with 5 ml of ice-cold Dulbecco’s PBS (FUJIFILM Wako Chemicals) two times and harvested with 250 μl of the TX114 lysis buffer (50 mM Tris–HCl pH 7.4, 150 mM NaCl, 5 mM MgCl_2_, 1% Triton X-114 (Sigma-Aldrich), Complete protease inhibitor cocktail EDTA-free, and 1 mM DTT) using a cell scraper. The lysates were cleared by centrifugation at 20,000*g* for 10 min at 4 °C, and the resulting supernatant was warmed up to 37 °C for 3 min, followed by centrifugation at 3000*g* for 5 min at 25 °C. The resulting upper phase was collected as the aqueous phase and the oily droplet at the bottom of the tube as the detergent phase, and each was washed once as follows. The aqueous phase was collected into a new tube and supplemented with 1% Triton X-114, and the oily droplet at the bottom of the tube was supplemented with the aforementioned buffer not containing Triton X-114. They were incubated on ice for 5 min to give a clear solution and then warmed up, centrifuged, and collected as described previously. Finally, Triton X-114 and buffer were added, respectively, to the aqueous and detergent phases to obtain equal volumes and approximately the same Triton X-114 content for both samples. Proper fractionation by hydrophobicity was validated by immunoblotting using antibodies against DJ-1 (hydrophilic) and the transferrin receptor (hydrophobic).

### Immunoblotting and immunoprecipitation

Immunoblotting of cell lysates was performed as described previously (37). Briefly, cells were lysed with the lysis buffer (50 mM Tris–HCl pH 7.5, 1 mM EGTA, 50 mM sodium fluoride, 10 mM β-glycerophosphate, 5 mM sodium pyrophosphate, 0.27 M sucrose, 1%(v/v) Triton X-100, 1 mM sodium orthovanadate, 0.1 μg/ml microcystin-LR (FUJIFILM Wako chemicals), and Complete protease inhibitor cocktail EDTA-free), and the lysates were cleared by centrifugation at 20,000*g* for 10 min at 4 °C. The protein concentration of the cleared lysates was determined by the Bradford assay and adjusted with the lysis buffer. Cell lysates as well as the fractionated samples were mixed with a concentrated SDS-PAGE sample buffer and boiled at 100 °C for 5 min before loading onto SDS-PAGE gels.

For immunoprecipitation, cell lysates were prepared as described previously, and the protein concentration of the lysates was adjusted to 1.0 mg/ml with the lysis buffer. Twenty microliters (bed volume) of preequilibrated HA-agarose (Sigma-Aldrich; A2095) was added to 500 μl of the lysate, and the mixture was rotated at 4 °C for 2 h. The beads were spun down by centrifugation at 2500*g* for 30 s at 4 °C and washed with the ice-cold lysis buffer three times. After carefully removing the wash buffer using a syringe needle, bound proteins were eluted by adding 1× SDS-PAGE sample buffer not containing BME, followed by boiling at 100 °C for 5 min. The beads were removed by passing through an empty spin column (Bio-Rad Inc; #7326204), and the flow-through was supplemented with BME at a final concentration of 1% (v/v).

### Immunocytochemistry

Cells were seeded on noncoated glass coverslips. On the next day, cells were fixed with 4% paraformaldehyde dissolved in PBS for 15 min at room temperature. Cells were then washed with PBS three times and permeabilized with PBS containing 0.1% (v/v) Triton X-100 for 30 min at room temperature. Cells were washed with PBS three times and blocked with PBS containing 3% bovine serum albumin for 30 min at room temperature. Later, cells were incubated with primary antibodies diluted in the blocking solution at 4 °C overnight. Cells were then washed with PBS three times and incubated with fluorescently labeled secondary antibodies diluted in the blocking solution for 1 h at room temperature in the dark. Cells were extensively washed with PBS in the dark and mounted on a slide glass with a ProLong Diamond Antifade Mountant (Thermo Fisher Scientific) overnight at room temperature. Mounted samples were observed under a confocal laser-scanning microscope (SP5; Leica Microsystems). Image manipulation was done on ImageJ (https://imagej.nih.gov/ij/). Antibodies used for immunocytochemistry are listed in Table S3.

### Statistical analysis

Statistical significance of the difference was calculated using GraphPad Prism 8 (GraphPad Software; https://www.graphpad.com/). Tests used for examining statistical significance are described in the figure legends. The *p*-values below 0.05 were considered to be statistically significant. All *p*-values along with the statistical tests used are reported in Table S1.

## Data availability

All supporting data are included in the main article and its Supporting Information

## Supporting information

This article contains supporting information.

## Conflict of interest

The authors declare that they have no conflicts of interest with the contents of this article.
