# Supplementary material for: The atypical Rab GTPase associated with Parkinson’s disease, Rab29, is localized to membranes
Source: J Biol Chem. 2022 Sep 16;298(10):102499. doi: 10.1016/j.jbc.2022.102499 (PMC9574512; doi:10.1016/j.jbc.2022.102499)
Supplement: Supplemental Tables S1–S5 [file mmc1.docx]

# Table S1.

A summary of the p-values and statistical tests used in the figures.

| *Figure* | *Comparison* | *P-value* | *Test* |
| --- | --- | --- | --- |
| 1B | Rab10 vs Rab29 | 0.00499 (**) | Student’s t-test (unpaired, two-tailed) |
| 1D | Rab10 vs Rab29 (brain) | 0.00603 (**) | Two-way ANOVA (Sidak’s test) |
|  | Rab10 vs Rab29 (heart) | <0.00001 (***) |  |
|  | Rab10 vs Rab29 (lung) | 0.00017 (***) |  |
|  | Rab10 vs Rab29 (liver) | 0.00001 (***) |  |
|  | Rab10 vs Rab29 (kidney) | 0.10231 |  |
|  | Rab10 vs Rab29 (spleen) | 0.00069 (***) |  |
| 2B | empty vs GDI1 at 10 nM (Rab10) | 0.90127 | Two-way ANOVA (Tukey’s test) |
|  | empty vs GDI1 at 10 nM (Rab29) | 0.99662 |  |
|  | empty vs GDI1 at 30 nM (Rab10) | 0.51379 |  |
|  | empty vs GDI1 at 30 nM (Rab29) | 0.99921 |  |
|  | empty vs GDI1 at 100 nM (Rab10) | 0.00554 (**) |  |
|  | empty vs GDI1 at 100 nM (Rab29) | 0.99993 |  |
|  | empty vs GDI1 at 300 nM (Rab10) | <0.00001 (***) |  |
|  | empty vs GDI1 at 300 nM (Rab29) | 0.99940 |  |
|  | empty vs GDI1 at 1000 nM (Rab10) | <0.00001 (***) |  |
|  | empty vs GDI1 at 1000 nM (Rab29) | 0.97839 |  |
| 2D | empty vs GDI2 at 10 nM (Rab10) | 0.99985 | Two-way ANOVA (Tukey’s test) |
|  | empty vs GDI2 at 10 nM (Rab29) | 0.97779 |  |
|  | empty vs GDI2 at 30 nM (Rab10) | 0.86631 |  |
|  | empty vs GDI2 at 30 nM (Rab29) | 0.98426 |  |
|  | empty vs GDI2 at 100 nM (Rab10) | 0.06944 |  |
|  | empty vs GDI2 at 100 nM (Rab29) | 0.89425 |  |
|  | empty vs GDI2 at 300 nM (Rab10) | <0.00001 (***) |  |
|  | empty vs GDI2 at 300 nM (Rab29) | 0.67676 |  |
|  | empty vs GDI2 at 1000 nM (Rab10) | <0.00001 (***) |  |
|  | empty vs GDI2 at 1000 nM (Rab29) | 0.35787 |  |
| 3C | Rab3A vs Rab29 | 0.00037 (**) | One-way ANOVA (Dunnett’s test) |
|  | Rab5 vs Rab29 | 0.00063 (***) |  |
|  | Rab7A vs Rab29 | 0.01169 (*) |  |
|  | Rab8A/B vs Rab29 | 0.01630 (*) |  |
|  | Rab10 vs Rab29 | 0.00052 (***) |  |
|  | Rab12 vs Rab29 | 0.04153 (*) |  |
|  | Rab32 vs Rab29 | 0.05307 |  |
| 3D | Rab3A: WT vs DKO | 0.00188 (**) | Two-way ANOVA (Tukey’s test) |
|  | Rab3A: DKO vs DKO + V5-GDI1 | 0.00489 (**) |  |
|  | Rab5: WT vs DKO | <0.00001 (***) |  |
|  | Rab5: DKO vs DKO + V5-GDI1 | 0.00056 (***) |  |
|  | Rab7A: WT vs DKO | 0.00302 (**) |  |
|  | Rab7A: DKO vs DKO + V5-GDI1 | 0.00117 (**) |  |
|  | Rab8A/B: WT vs DKO | 0.04623 (*) |  |
|  | Rab8A/B: DKO vs DKO + V5-GDI1 | 0.15612 |  |
|  | Rab10: WT vs DKO | 0.00129 (**) |  |
|  | Rab10: DKO vs DKO + V5-GDI1 | 0.00819 (**) |  |
|  | Rab12: WT vs DKO | 0.46936 |  |
|  | Rab12: DKO vs DKO + V5-GDI1 | 0.50143 |  |
|  | Rab29: WT vs DKO | 0.74632 |  |
|  | Rab29: DKO vs DKO + V5-GDI1 | 0.17629 |  |
|  | Rab32: WT vs DKO | 0.03028 (*) |  |
|  | Rab32: DKO vs DKO + V5-GDI1 | 0.00334 (**) |  |
| 6B | Rab10 (Membrane) vs Rab29 (Membrane) | 0.02930 (*) | One-way ANOVA (Tukey’s test) |
| 6D | Rab10 (Membrane) vs Rab29 (Membrane) | 0.00036 (***) | One-way ANOVA (Tukey’s test) |
| 6F | Rab10 (Membrane) vs Rab29 (Membrane) | 0.00085 (***) | One-way ANOVA (Tukey’s test) |
| 7C | Rab10: 0 μM vs 5 μM | 0.63266 | One-way ANOVA (Dunnett’s test) |
|  | Rab10: 0 μM vs 10 μM | 0.01526 (*) |  |
|  | Rab29: 0 μM vs 5 μM | 0.56737 | One-way ANOVA (Dunnett’s test) |
|  | Rab29: 0 μM vs 10 μM | 0.01786 (*) |  |
| 8C | Rab10: sgEmpty vs sg*RABGGTA* | 0.00707 (**) | Student’s t-test (unpaired, two-tailed) |
|  | Rab29: sgEmpty vs sg*RABGGTA* | 0.02469 (*) | Student’s t-test (unpaired, two-tailed) |
| 8F | Rab10: sgEmpty vs sg*RABGGTB* | 0.00073 (***) | Student’s t-test (unpaired, two-tailed) |
|  | Rab29: sgEmpty vs sg*RABGGTB* | 0.00509 (**) | Student’s t-test (unpaired, two-tailed) |
| 9B | Rab10 WT vs Rab29 WT | 0.08877 | One-way ANOVA (Dunnett’s test) |
|  | Rab29 WT vs Rab29 CCSS | 0.00162 (**) |  |
|  | Rab29 WT vs Rab29 D63A | 0.01278 (*) |  |
| 9D | Rab10 WT vs Rab29 WT | 0.00262 (**) | One-way ANOVA (Dunnett’s test) |
|  | Rab29 WT vs Rab29 CCSS | <0.0001 (***) |  |
|  | Rab29 WT vs Rab29 D63A | 0.00333 (*) |  |
| 9G | Rab29 vs Rab29+10 | 0.00068 (***) | Student’s t-test (unpaired, two-tailed) |
| S1B | Rab10-NT vs Rab10-CHX | 0.80348 | One-way ANOVA  (Tukey’s test) |
|  | Rab10-NT vs Rab29-NT | 0.00045 (***) |  |
|  | Rab10-CHX vs 29-CHX | 0.00094 (***) |  |
|  | Rab29-NT vs Rab29-CHX | 0.99811 |  |
| S3B | Rab10 vs Rab29 (siNT) | <0.00001 (***) | Two-way ANOVA (Sidak’s test) |
|  | Rab10 vs Rab29 (siRABAC1) | 0.00030 (***) |  |
|  | Rab10 vs Rab29 (siPRAF2) | <0.00001 (***) |  |
|  | Rab10 vs Rab29 (siARL6IP5) | 0.00002 (***) |  |
|  | siNT vs siRABAC1 (Rab10) | 0.34500 | One-way ANOVA (Dunnett’s test) |
|  | siNT vs siPRAF2 (Rab10) | 0.99938 |  |
|  | siNT vs siARL6IP5 (Rab10) | 0.92570 |  |
|  | siNT vs siRABAC1 (Rab29) | 0.64133 | One-way ANOVA (Dunnett’s test) |
|  | siNT vs siPRAF2 (Rab29) | 0.84911 |  |
|  | siNT vs siARL6IP5 (Rab29) | 0.66671 |  |

# Table S2.

A list of the reported localization of WT and mutant Rab29 which were shown by immunocytochemistry in the past studies and this study.

|  | **Cells** | **Transfection** | **WT** | **Q67L**  **(GTP-bound)** | **T21N**  **(GDP-bound)** | **D63A**  **(nucleotide-free)** |
| --- | --- | --- | --- | --- | --- | --- |
| This study | U2OS | Stable | TGN+ER? | TGN | Cytosol | TGN |
| Spanò et al., PNAS, 2011 | HeLa | Transient | Golgi | N.D. | N.D. | N.D. |
| MacLeod et al., Neuron, 2013 | SH-SY5Y | Transient | Golgi | Golgi+cytosol | Cytosol | N.D. |
| Wang et al., PLOS ONE, 2014 | NRK, HeLa | Endogenous | Golgi+cytosol | N.A. | N.A. | N.A. |
|  | HeLa | Transient | TGN+cytosol | Cytosol | Cytosol | N.D. |
| Beilina et al., PNAS, 2014 | ? | Transient | TGN | Cytosol | Cytosol | N.D. |
| Fujimoto et al., BBRC, 2018 | HEK293 | Transient | TGN | Cytosol | Cytosol | N.D. |
| Liu et al., HMG, 2018 | SH-SY5Y | Transient | Golgi? | Cytosol | Golgi?+cytosol | N.D. |
| Madero-Perez et al., Frontiers Mol Neurosci, 2018 | HEK293T | Transient | TGN | TGN+cytosol | TGN+cytosol | N.D. |

N.D.: not determined, N.A.: not applicable.

# Table S3.

A list of antibodies used in this study with their suppliers and dilutions for immunoblotting and immunocytochemistry.

| Antigen (Clone name) | Supplier (Catalog number) | Dilution for immunoblotting (IB) or immunocytochemistry (ICC) |
| --- | --- | --- |
| DJ-1 | AlphaGenix (#24369) | 1:1000 (IB) |
| FLAG tag (M2) | Sigma-Aldrich (F3165-1MG) | 1:1,000 (IB) |
| GAPDH (6C5) | Santa Cruz Biotechnology (sc-32233) | 0.1 μg/mL (IB) |
| GDI1/2 (1H3) | Sigma-Aldrich (WH0002664M8-100UG) | 1 μg/mL (IB) |
| GFAP | BioLegend (829401) | 1:1000 (ICC) |
| HA tag (3F10) | Roche (11867423001) | 0.1 μg/mL (IB)  0.5 μg/mL (ICC) |
| HA tag | MBL (561) | 30 μg/mL (ICC) |
| Iba1 | Fujifilm Wako (019-19741) | 1:500 (ICC) |
| Na+/K+ ATPase α-1 subunit | Developmental Studies Hybridoma Bank (a6F) | 0.2 μg/mL (IB) |
| p53 | Santa Cruz Biotechnology (sc-126) | 0.4 μg/mL (IB) |
| Rab3A (D7B10) | Cell Signaling Technology (#12214) | 1:1,000 (IB) |
| Rab5 (C8B1) | Cell Signaling Technology (#3547) | 1:1,000 (IB) |
| Rab7A (EPR7589) | Abcam (ab137029) | 1 μg/mL (IB) |
| Rab8A/B (4/Rab8) | Becton Dickinson (610845) | 1:1,000 (IB) |
| Rab10 (MJF-R23) | Abcam (ab237703) | 1 μg/mL (IB) |
| Rab10 (D36C4) | Cell Signaling Technology (#8127) | 1:1,000 (IB) |
| Rab12 | Proteintech (18843-1-AP) | 1:500 (IB) |
| Rab29 (MJF-R30-124) | Abcam (ab256526) | 1 μg/mL (IB) |
| Rab32 (D-11) | Santa Cruz Biotechnology (sc-377472) | 1:500 (IB) |
| RABGGTA (B-9) | Santa Cruz Biotechnology (sc-393545) | 1:500 (IB) |
| RABGGTB (B-8) | Santa Cruz Biotechnology (sc-365926) | 1:500 (IB) |
| TGN46 | Bio-Rad (AHP500G) | 0.5 μg/mL (ICC) |
| Transferrin receptor | BD Transduction Laboratories (612124) | 1:1000 (IB) |
| V5 tag | Thermo Fisher Scientific (R960-25) | 1:1,000 (IB) |
| V5 tag | Abcam (ab9137) | 2 μg/mL (ICC) |

# Table S4.

Nucleotide sequences of primers and a cDNA library used for cloning genes.

| Genes | cDNA library | Sequence (5’ -> 3’) |
| --- | --- | --- |
| human *GDI1* | human brain | TGAGCGGCCGCGATGGACGAGGAATACGATGTGATC |
|  |  | TGAGGTACCTCACTGCTCAGCTTCTCCAAAG |
| human *GDI2* | human brain | ATGAATGAGGAGTACGACGTGATCG |
|  |  | TTAGTCTTCCCCATAGATGTCATTCTTCTTGC |

# Table S5.

Guide RNA (gRNA) sequences used for generating knockout cells. Note that two gRNAs (left and right) were simultaneously used for knocking out a gene, as we used Cas9 nickase (D10A) which requires two adjacent gRNAs for cleaving target regions. For target sequences not having a “G” at the 5’ end, a “G” was added to the 5’ end of the sequences for efficient transcription. PAM motifs are highlighted in yellow. Intron sequences are underlined.

| Target gene (position) | Left (5’ to 3’) | Right (5’ to 3’) |
| --- | --- | --- |
| *GDI1*  (intron 4 - exon 5) | AAACATGCCCATCAGATCTAAGG | ACGGCGCTTCCGCAAGTTCCTGG |
| *GDI2*  (exon 5) | CGAAGTTGGCAACATACACTAGG | AAAGATCCAAGAACTTTTGAAGG |
| *RABGGTA*  (intron 1 - exon 2) | TGAACGGGTTGGAAGAGTGCAGG | CTTGTAGCACGGACGCCTGAAGG |
| *RABGGTA*  (intron 2 - exon 3) | CAGCCTGGCGCTAAGAAGATAGG | TGAGCTGGATGAGTCCGTGCTGG |
| *RABGGTA*  (exon 4) | AAGACTTGGGGTTCACCCGCAGG | GTACCTGGCACCACCGATGCTGG |
| *RABGGTB*  (exon 1 - intron 2) | TCACACTTACCATGTCTAACAGG | GTTTAGCGCTGCTGTCCGGATGG |
| *RABGGTB*  (exon 6 - intron 7) | ATAAATTACCTGCCCAGCATGGG | ATGAAAATGTATTGTCATTTTGG |
| *RABGGTB*  (exon 8) | CACCATGAGTAGCATACATCTGG | CTGGCTTCCCTAAAGATAATTGG |
