# Supplementary material for: The atypical Rab GTPase associated with Parkinson’s disease, Rab29, is localized to membranes
Source: J Biol Chem. 2022 Sep 16;298(10):102499. doi: 10.1016/j.jbc.2022.102499 (PMC9574512; doi:10.1016/j.jbc.2022.102499)
Supplement: Supplemental Figures S1–S5 [file mmc2.docx]

# Figure S1.


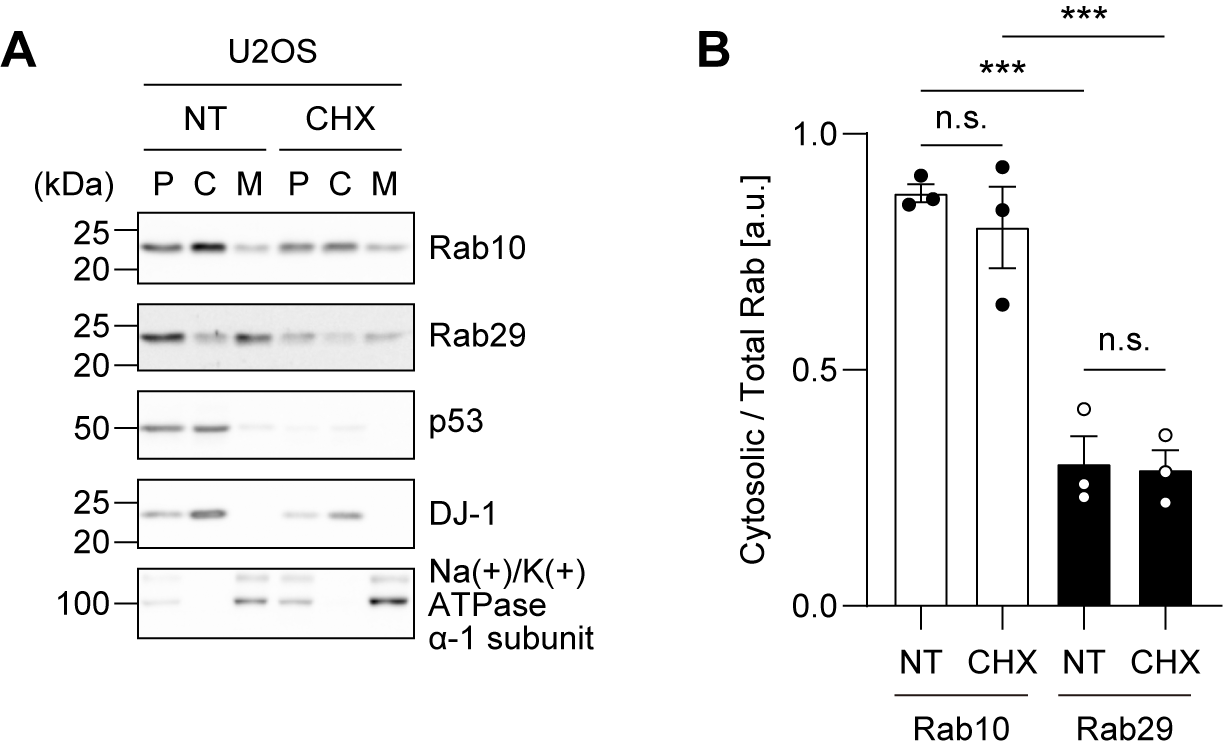


## The effect of blocking protein synthesis of the membrane localization of Rab10 and Rab29.

(A) Post-nuclear supernatants (P) of U2OS Flp-In T-REx cells treated with 100 μg/mL cycloheximide for 0 (non-treat; NT) or 48 h (CHX) were fractionated into the cytosol (C) and membrane (M) fractions by ultracentrifugation. Representative immunoblots of three independent experiments done in triplicate with the indicated antibodies are shown. p53 is a representative of a rapidly degrading protein, showing the inhibitory effect of cycloheximide on protein synthesis. DJ-1 was used as a representative cytosolic protein, whereas Na+/K+ ATPase α-1 subunit was used as a membrane protein.

(B) The band intensity of the Rab proteins in (A) was quantified, and the ratio of cytosolic Rab to total Rab (cytosol + membrane) was calculated. The circles, the bars, and the error bars in the graph represent the individual values, the mean values, and their standard errors, respectively. The p-values and statistical tests used are summarized in Table S1.

# Figure S2


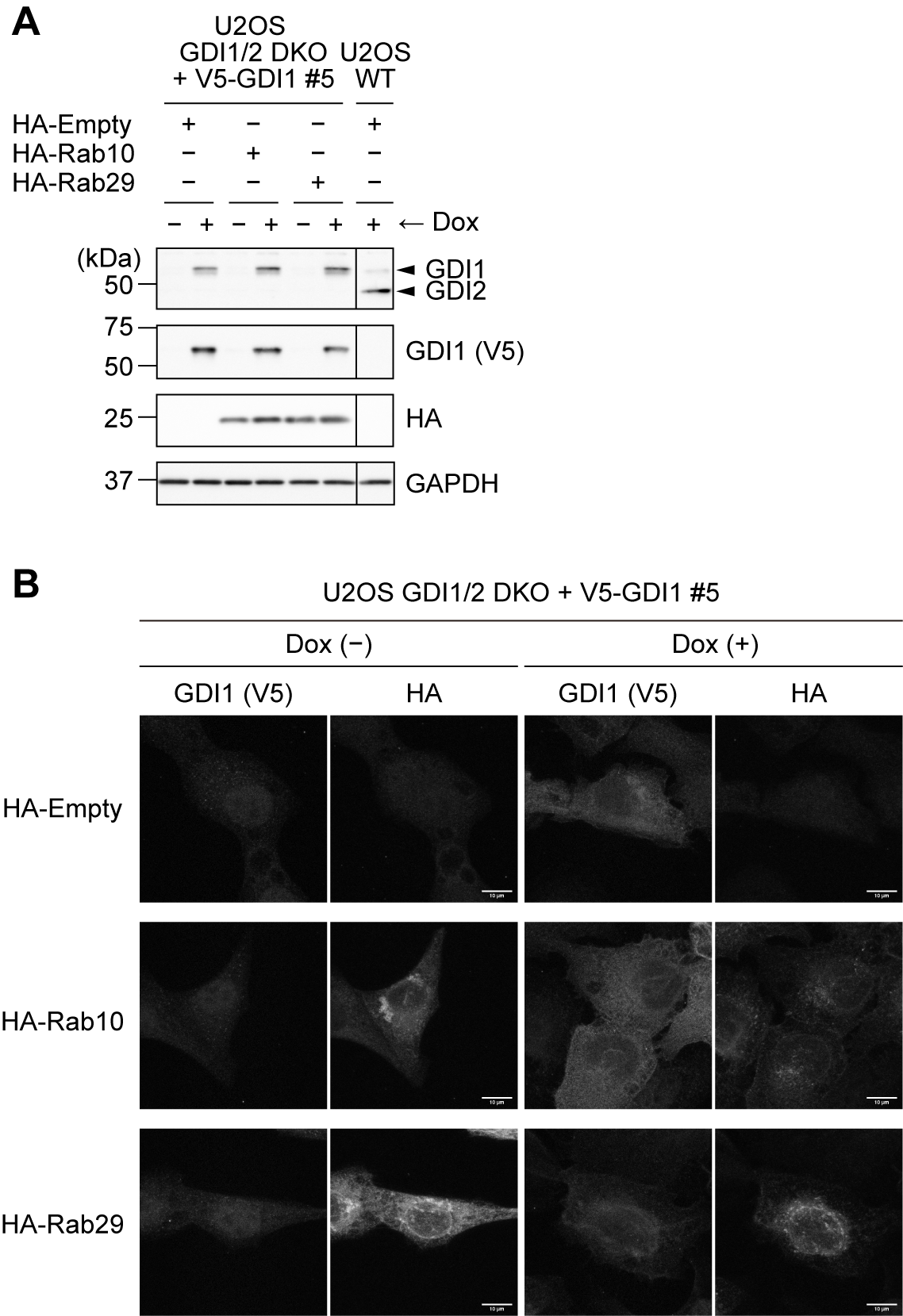


## The subcellular localization of Rab10 and Rab29 in the presence or absence of GDIs.

(A) U2OS GDI1/2 DKO + V5-GDI1 #5 cells were stably transfected with HA-Empty, HA-Rab10, or HA-Rab29. Where indicated, cells were treated with dox to induce the expression of V5-GDI1. The same amount of cell lysates prepared from U2OS WT cells stably overexpressing HA-Empty was run on the same gel as a positive control for the immunoblotting of GDI1/2. The solid line in the immunoblot indicates that the lane of molecular weight markers has been clipped. Immunoblots with the indicated antibodies are shown. GAPDH was used as a loading control.

(B) Immunocytochemistry (ICC) of the cells shown in (A) with anti-V5 and anti-HA antibodies. Scale bars: 10 µm.

# Figure S3.


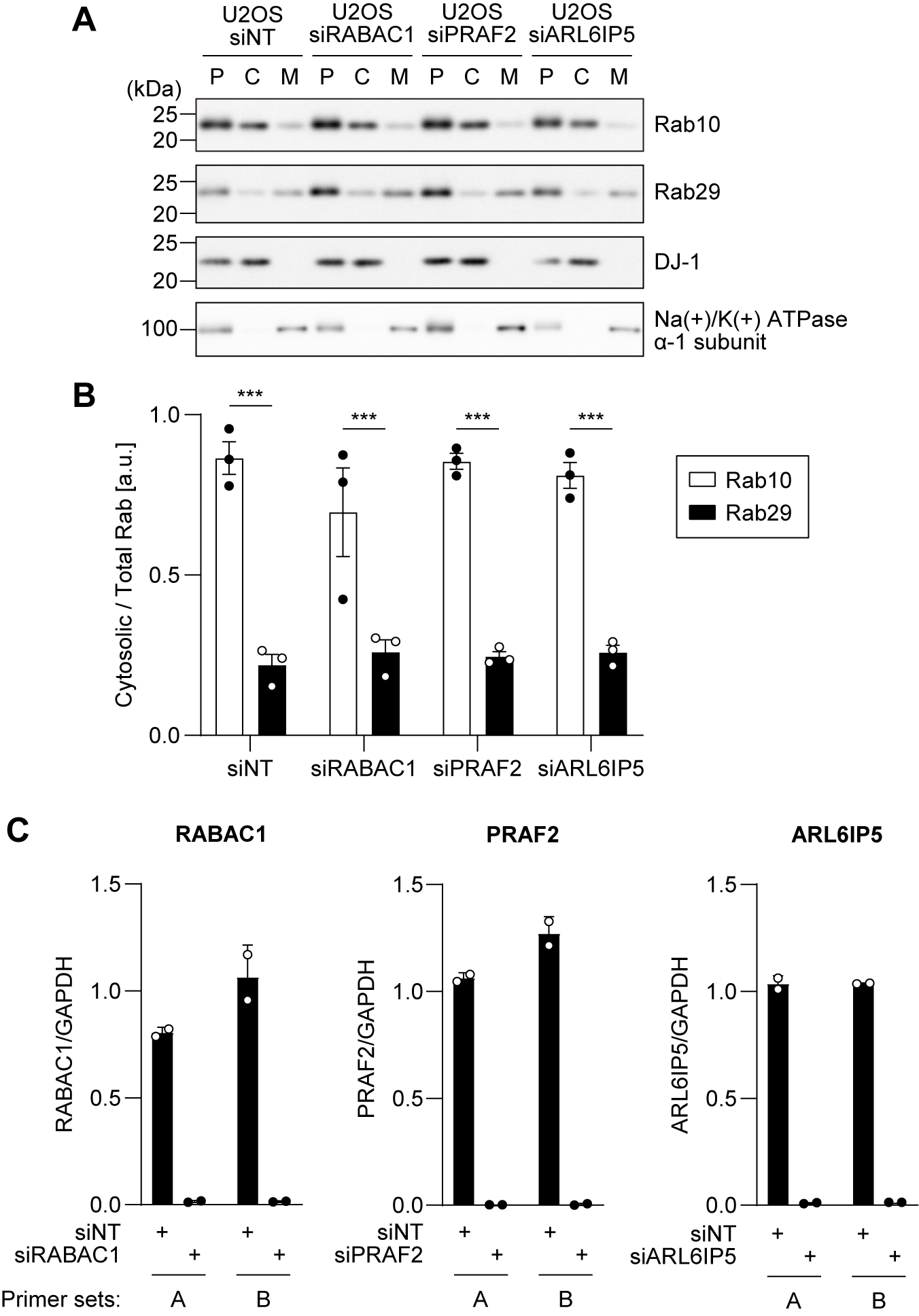


## The effect of the knockdown of the PRAF family proteins on the membrane localization of Rab10 and Rab29.

(A) U2OS cells were transfected with siRNAs targeting no genes (non-target; siNT), RABAC1 (siRABAC1), PRAF2 (siPRAF2), or ARL6IP5 (siARL6IP5), and their post-nuclear supernatants (P) were fractionated into the cytosol (C) and membrane (M) fractions by ultracentrifugation. Representative immunoblots of three independent experiments with the indicated antibodies are shown. DJ-1 was used as a representative cytosolic protein, whereas Na+/K+ ATPase α-1 subunit was used as a membrane protein.

(B) The band intensity of the Rab proteins was quantified, and the ratio of cytosolic Rab to total Rab (cytosol + membrane) was calculated. The circles, the bars, and the error bars in the graphs represent individual values, the mean values, and their standard errors, respectively. The p-values and statistical tests used are summarized in Table S1.

(C) The amounts of mRNAs of RABAC1, PRAF2, and ARL6IP5 were quantified by reverse transcription-quantitative PCR (RT-qPCR) using two independent primer sets (A and B). The crossing point (Cp) values for each gene were normalized by that for GAPDH. The experiment was carried out in duplicate having siNT (NT) as a negative control. The circles and the error bars in the graph represent individual values and the standard deviation, respectively.

# Figure S4.


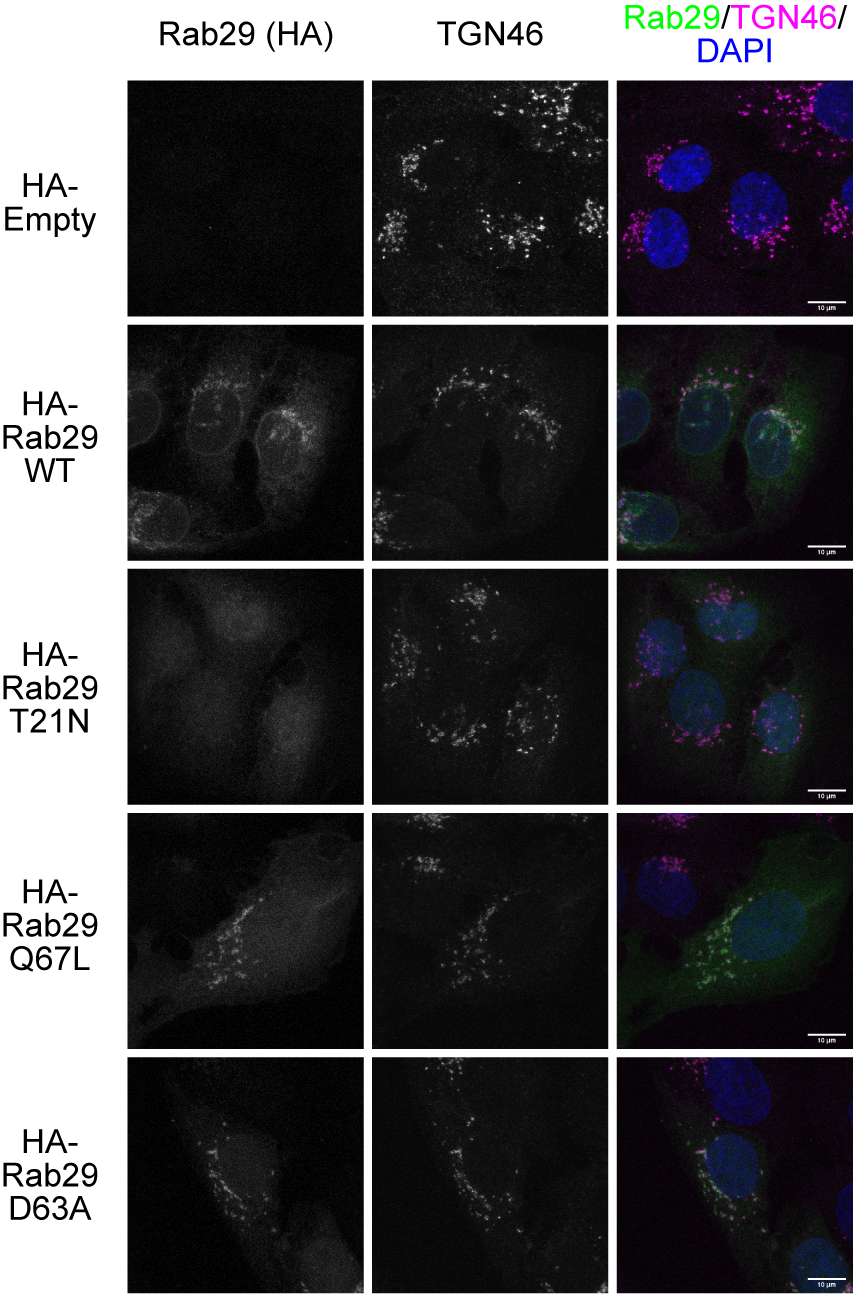


## The subcellular localization of Rab29 harboring mutations mimicking the GDP-bound, GTP-bound, and nucleotide-free forms.

U2OS cells stably overexpressing HA-Empty, HA-Rab29 WT, HA-Rab29 T21N, HA-Rab29 Q67L, or HA-Rab29 D63A were immunostained with anti-HA and anti-TGN46 antibodies. The nuclei were stained with DAPI. In the merged images, HA, TGN46, and the DAPI staining are shown in green, magenta, and blue, respectively. Scale bars: 10 µm.

# Figure S5.


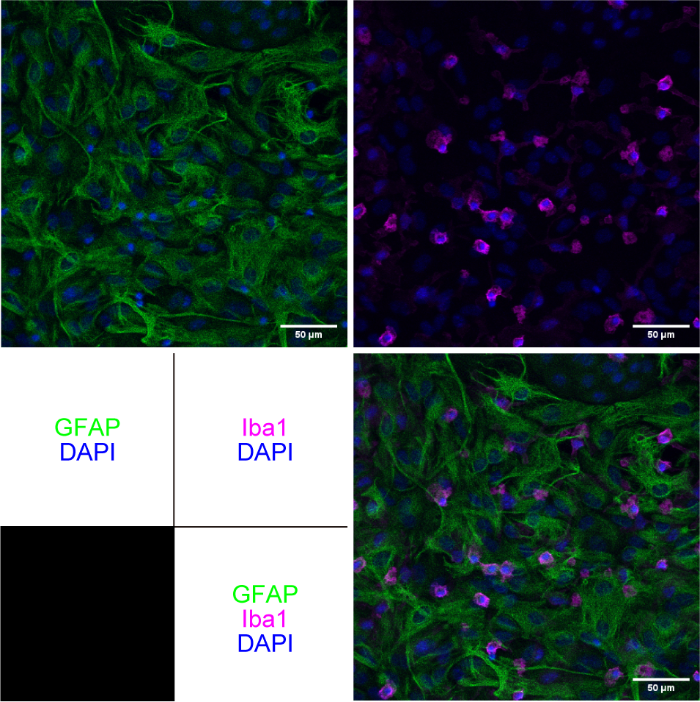


## The composition of mixed glial culture examined by immunocytochemistry

The mixed glial culture prepared from a postnatal-day-2 (P2) mouse brain was stained with anti-GFAP (activated astrocytic marker; green) and anti-Iba1 (microglial marker; magenta) antibodies. Nuclei were stained with DAPI (blue). Approximately 66% of the culture was comprised of astrocytes, 25% was microglia, and 9% was negative for both markers.
